# Phenotypic plasticity of *Escherichia coli* upon exposure to physical stress induced by ZnO nanorods

**DOI:** 10.1038/s41598-019-44727-w

**Published:** 2019-06-12

**Authors:** Kinga Matuła, Łukasz Richter, Marta Janczuk-Richter, Wojciech Nogala, Mikołaj Grzeszkowiak, Barbara Peplińska, Stefan Jurga, Elżbieta Wyroba, Szymon Suski, Henryk Bilski, Adrian Silesian, Hans A. R. Bluyssen, Natalia Derebecka, Joanna Wesoły, Joanna M. Łoś, Marcin Łoś, Przemyslaw Decewicz, Lukasz Dziewit, Jan Paczesny, Robert Hołyst

**Affiliations:** 1 Institute of Physical Chemistry of the Polish Academy of Sciences, Kasprzaka 44/52, 01-224 Warsaw, Poland; 2 Nanobiomedical Centre, Adam Mickiewicz University, Umultowska 85, 61-614 Poznań, Poland; 3 Nencki Institute of Experimental Biology of the Polish Academy of Sciences, Pasteur 3, 02-093 Warsaw, Poland; 4 Department of Human Molecular Genetics, Institute of Biotechnology and Molecular Biology, Faculty of Biology, Adam Mickiewicz University, Umultowska 89, 61-614 Poznań, Poland; 5 Laboratory of High Throughput Technologies and Department of Human Molecular Genetics, Institute of Biotechnology and Molecular Biology, Faculty of Biology, Adam Mickiewicz University, Umultowska 89, 61-614 Poznań, Poland; 6 Department of Molecular Genetics of Bacteria, Faculty of Biology, University of Gdańsk, Wita Stwosza 59, 80-308 Gdańsk, Poland; 7 Phage Consultants, Partyzantów 10/18, 80-254 Gdańsk, Poland; 8 Department of Bacterial Genetics, Institute of Microbiology, Faculty of Biology, University of Warsaw, Miecznikowa 1, 02-096 Warsaw, Poland

**Keywords:** Molecular biology, Environmental microbiology

## Abstract

Evolution of bacteria to selective chemical pressure (e.g. antibiotics) is well studied in contrast to the influence of physical stressors. Here we show that instantaneous physical stress in a homogeneous environment (without concentration gradient) induces fast adaptation of *Escherichia coli*. We exposed *E*. *coli* to a large number of collisions of around 10^5^ per bacterium per second with sharp ZnO nanorods. The pressure exerted on the bacterial cell wall was up to 10 GPa and induced phenotype changes. The bacteria’s shape became more spherical, the density of their periplasm increased by around 15% and the average thickness of the cell wall by 30%. Such *E*. *coli* cells appeared almost as Gram-positive bacteria in the standard Gram staining. Additionally, we observed a combination of changes occurring at the genomic level (mutations identified in form of single nucleotide polymorphisms) and down-regulation of expression of 61 genes encoding proteins involved in β-oxidation of fatty acids, glycolysis, the citric acid cycle, as well as uptake of amino acids and enzyme cofactors. Thus, we show that bacteria undergo phenotypic changes upon instantaneous, acute physical stress without any obviously available time for gradual adaptation.

## Introduction

In their natural environment, bacterial cells evolve, however, bringing the evolutionary process into the laboratory allows for tracking changes in genome and phenotype in a more controlled manner^1–3^. Although, the natural evolution of microorganism is of great importance since we can gain new insight into mutation rate and the correlation between the genomic and phenotypic plasticity, currently it is the evolution of bacteria under antibiotic pressure that takes on increased importance. The real threat of “post-antibiotic” era forced the scientific community to intensify studies in this field^4–8^ since bacteria develop resistance to antibiotic selective pressure in a heterogeneous environment (e.g. gradient plates with antibiotics) even within hours^9^. Nanoparticles, that recently have become promising agents in the fight against multi-drug-resistant bacteria, could replace antibiotics in some applications^10–15^. However, there are alarming reports published on resistance against silver nanoparticles, as released silver ions can act similarly to antibiotics, including the acquisition of resistance^16–19^. In fact, nanoparticles can be perceived both, as chemical and physical factors influencing bacteria functioning, depending on the nature of interactions between nanomaterials and bacterial cells^20,21^. Hopes are high, especially regarding nanostructures and their non-chemical mechanisms of killing bacteria, e.g. contact killing^11,22,23^ or mechanical puncturing^24–26^. Nevertheless, the influence of physical stress induced by nanoparticles on the adaptation of bacteria still remains elusive and lacks understanding, in contrast to deeply analyzed effects of antibiotic treatments. Therefore, it is important to investigate not only the effect of nanoparticles onto bacterial cells functioning (e.g. biofilm formation) and viability, but also changes caused by them at the cellular, genomic and transcriptomic levels. So far, no data have been demonstrated the real-time adaptation of bacteria upon exposure to nanoparticles or any other physical stressor. Here, we provide evidence for fast adaptation of *E*. *coli* to physical stress induced by nanoparticles. We exposed bacteria to collisions with sharp zinc oxide (ZnO) nanorods (NR) which exerted a high pressure at the surface of the bacteria. We observed that bacteria adapted to mechanical stress within hours. Additionally, our experiments were conducted in a homogeneous environment in contrast to the heterogeneous environment (with concentration gradient) used in studies on the acquisition of drug resistance by bacteria^4,9^.

## Results

### Exposure to ZnO nanorods

*Escherichia coli* were cultivated in a medium supplemented with ZnO nanorods. Zinc ions have limited influence on *E*. *coli*, as Gram-negative bacteria possess a lipopolysaccharide (LPS) layer, which acts as toxic ions scavenger (see Supplementary Information^27^). We found that during 24-hour incubation of ZnO NR (1 mg ml^−1^) in the growth medium, ~38 µg ml^−1^ of free Zn^2+^ ions were released (~17 µg ml^−1^ in MiliQ water). We proved that even higher concentration of 50 µg ml^−1^ of Zn^2+^ ions, had limited impact on *E*. *coli*^27^. The large size of the nanorods (length 312 ± 171 nm and width 76 ± 21 nm) was chosen to minimize the toxic effect of nanostructure (smaller portion of matter exhibits higher cytotoxicity)^28,29^, and to exert sufficient pressure at the cell wall. The concentration of NR was 1 mg ml^−1^ which corresponds to a large number of collisions 10^5^ per bacterium per second. During stirring of the medium (200 rpm) the flow was turbulent with Reynolds number ~10^4^. The characteristic relative velocities of the bacteria and the nanorods were of the order of v ~ 0.2–1.8 m s^−1^. The interaction between the nanorods and the bacterium can be described using the Hertzian theory of collisions since both objects come into mechanical contact^30^. The pressure exerted at the surface of the bacterium varies from 0.1 MPa up to 10 GPa depending on the size of the tip of the ZnO nanorod and the collision geometry (more calculations provided in the Supplementary Information in section 2, see also Supplementary Fig. S4). The lowest pressure is comparable to the turgor pressure inside the bacteria (~0.3 MPa^31^) and the highest exceeds 400 times the Young modulus of the bacteria (~25 MPa^32^). Experiments performed with an AFM tip with a radius of 2 nm, showed that when the force acting on the cantilever was 10 nN (pressure ~3,000 MPa), the holes created by the tip spontaneously “healed” with no visible traces left on the surface of the *E*. *coli* cell^33^. Thus, in principle during exposure to the pressure exerted by ZnO nanorods, the bacteria may be hit or punctured many times without losing their viability.

### Phenotypic changes of bacteria exposed to ZnO nanorods

**Figure 1:**
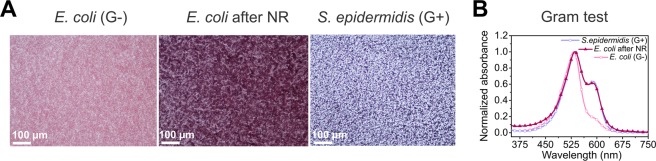
Gram staining of *E*. *coli* bacteria upon exposure to ZnO nanorods. (**A**) Direct images of Gram-stained bacteria obtained from optical microscope: control Gram-negative *E*. *coli* [*E*. *coli* (G−)], *E*. *coli* exposed to ZnO NR (marked as *E*. *coli* after NR) and control Gram-positive *S*. *epidermidis* [marked as *S*. *epidermidis* (G+)]. (**B**) Comparison of UV spectra of samples after Gram staining. The peak in the range about 530 nm corresponds to the presence of safranine. Maximum absorption for crystal violet that remains in the layer of peptidoglycan is around 590 nm.

The bacteria that survived the first treatment with ZnO NR were collected and exposed to nanorods in the second run of the 24-hour experiment (viability curves are shown in the Supplementary Information in section 3 and 4, see Supplementary Figs S5 and S6). It appeared that survivor *E*. *coli* became invulnerable to the mechanical stress, in this respect resembling Gram-positive bacteria^27^. This was also reflected in classical Gram staining, which depends on the amount of peptidoglycan within the cell wall of the bacteria. Figure 1A shows direct images after staining of the bacteria on glass slides showing the difference in the color of *E*. *coli* (control and exposed to nanorods) and *Staphylococcus epidermidis* (used as a referential Gram-positive strain). Figure 1B shows a quantitative interpretation of Gram staining based on UV-Vis spectra (see also Supplementary Fig. S7 in the Supplementary Information).

**Figure 2:**
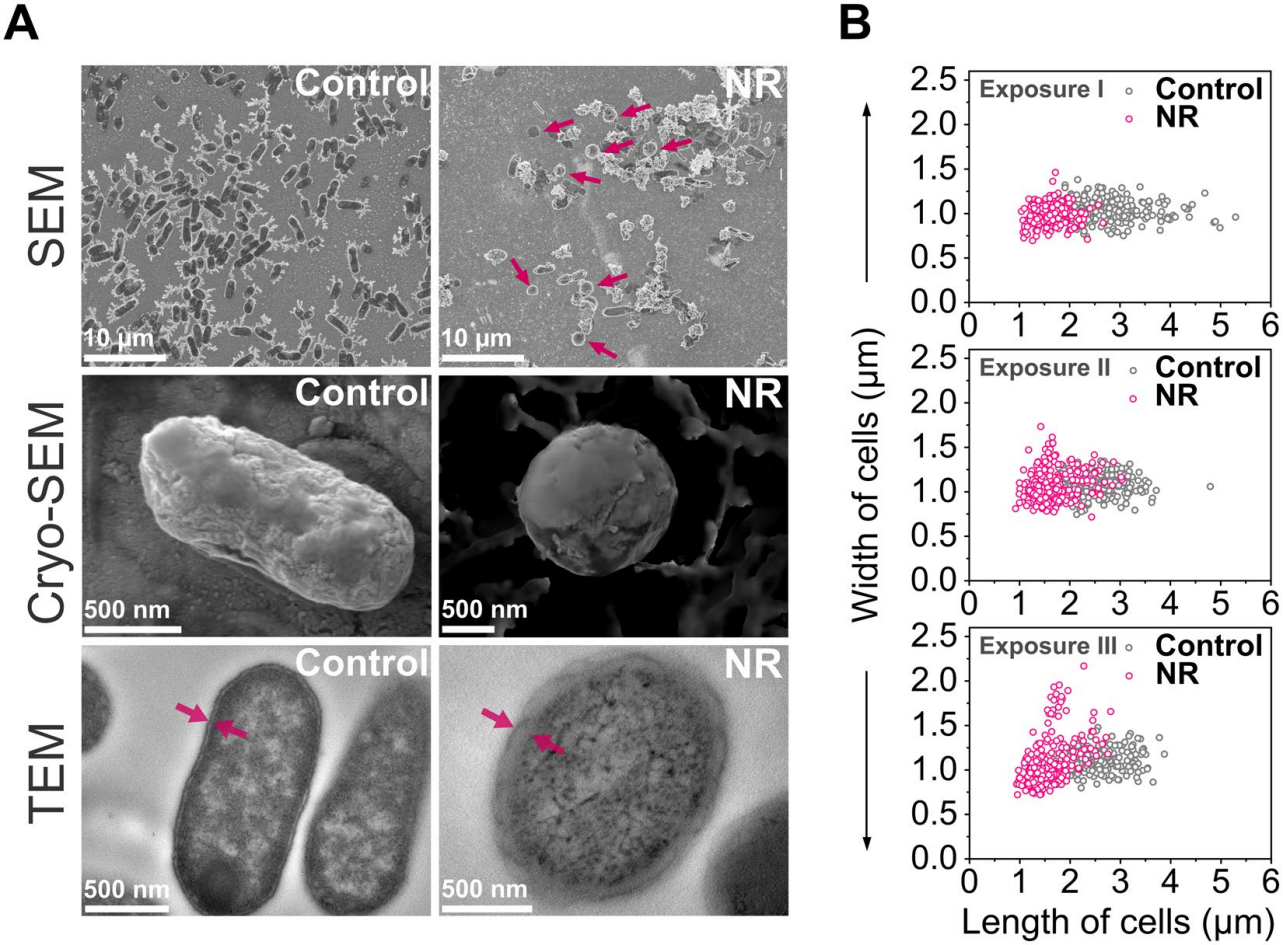
Change of phenotype of *E*. *coli* exposed to ZnO nanorods. (**A**) Images of non-treated *E*. *coli* (marked as Control) and cells exposed to ZnO nanorods (marked as NR) obtained by scanning electron microscopy (SEM), cryo-scanning electron microscopy (Cryo-SEM) and transmission electron microscopy (TEM). SEM images show non-treated samples of control bacteria and the bacteria after three exposures to ZnO nanorods. The arrows indicate spherical *E*. *coli* cells. Cryo-SEM images show the shape of control bacteria and the cells after a second exposure to nanorods. In the TEM images, the change in cell wall thickness of *E*. *coli* is indicated with arrows. (**B**) Change of width and length of the bacteria after the first, second and third exposure to nanorods. For each population at least 200 bacterial cells were examined. The number of spherical cells increased with each subsequent exposure to ZnO nanorods.

Also, the shape of *E*. *coli* cells changed after exposure to the nanorods. In addition to rod-like, elliptical and even spherical cells appeared in the samples (Fig. 2A). We observed the same phenotype changes in *Enterobacter aerogenes* (Gram-negative) but not in *Corynebacterium glutamicum* (Gram-positive). More experimental details and microscopy images are shown in the Supplementary Information in section 6 and Supplementary Fig. S8. Length and width of the control bacteria and the cells exposed to the nanorods were measured using scanning electron microscopy (SEM) after three, 24-hour each, exposures to nanorods (Fig. 1B). The average ratios of length to width of the control cells and the bacteria exposed to the nanorods after each run are shown in Supplementary Fig. S1 in the Supplementary Information. The length to width ratio decreased from 2.44 ± 0.52 before treatment (for native bacteria) to 1.48 ± 0.32 after 72 hours exposure to ZnO NR (***P < 0.001), more statistical data provided in Supplementary Fig. S1.

**Figure 3:**
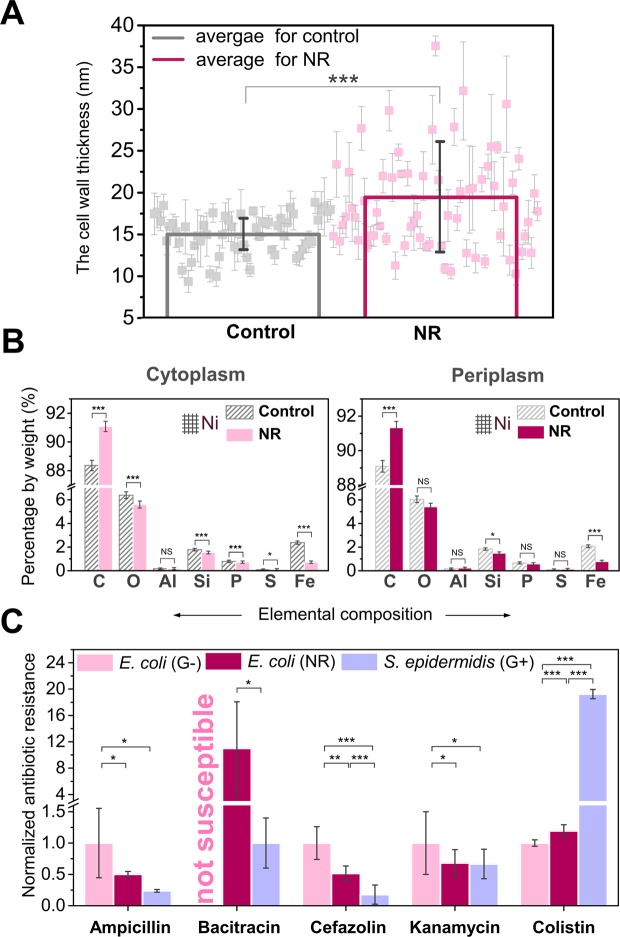
Change of the thickness and elemental composition of the cell wall of *E*. *coli* after exposure to ZnO nanorods render different susceptibility to antibiotics. (**A**) The average thickness of *E*. *coli* cell wall after the first exposure to ZnO nanorods (19.6 ± 6.7 nm) in comparison to the cell wall of the control cells (15.1 ± 2.4 nm) obtained by TEM. In the case of treated bacteria, one value (47.5 nm) is not shown to retain the transparency of presentation of all other data. (**B**) Elemental composition of the periplasm and the interior of *E*. *coli* after one exposure and the control cells analyzed by energy dispersive X-ray spectroscopy (EDS) on the nickel grids. (**C**) Minimal inhibitory concentration (MIC) for ampicillin, bacitracin, cefazolin, colistin and tetracycline determined for *E*. *coli* after exposure to ZnO nanorods. Error bars show standard error of the mean (s.e.m.), where *P < *0*.*05*; **P < *0*.*01*; ***P < *0*.*001*.

The average thickness of the cell wall of *E*. *coli* after one exposure to nanorods increased by 30% on average (***P < 0.001) (see Fig. 3A, more results are also provided in Supplementary Fig. S2 and in Supplementary Fig. S9 in the Supplementary Information in section 7). However, bacteria with up to a 215% increase in cell wall thickness were also found in the population. Changes in the elemental composition of the periplasm and the interior of the bacteria before and after exposure to the nanorods were also observed (see Figs 3B and S3). Based on these measurements we also deduced that the density of periplasm increased by around 15% in the case of ZnO NR-treated bacteria. The estimation was based on carbon content before and after exposure to ZnO NR. EDS collects data from the fixed volume of the sample. Since the structural building blocks are mainly composed of carbon, a higher amount of this element in a given volume translates to more structural constituents, i.e. higher density.

We checked whether a thicker cell wall could change the resistance of the bacteria to antibiotics. The minimum inhibitory concentration (MIC) for colistin, that acts as a surface active agent, increased by 19%. In contrast, increased susceptibility to antibiotics affecting bacterial cell wall synthesis (ampicillin, cefazolin), and protein synthesis (kanamycin, tetracycline) was observed in the case of the bacteria exposed to nanorods in comparison to control *E*. *coli* (Fig. 3C). Moreover, *E*. *coli* after the second exposure to nanorods became susceptible to bacitracin, while normally this antibiotic is used exclusively for the treatment of infections caused by Gram-positive bacteria. In general, exposure of *E*. *coli* to NR resulted in changes in the susceptibility to the majority of the studied antibiotics approaching MIC values characteristic for Gram-positive bacteria.

### SNP mutations identified in the genome of *E*. *coli* exposed to ZnO nanorods

**Figure 4:**
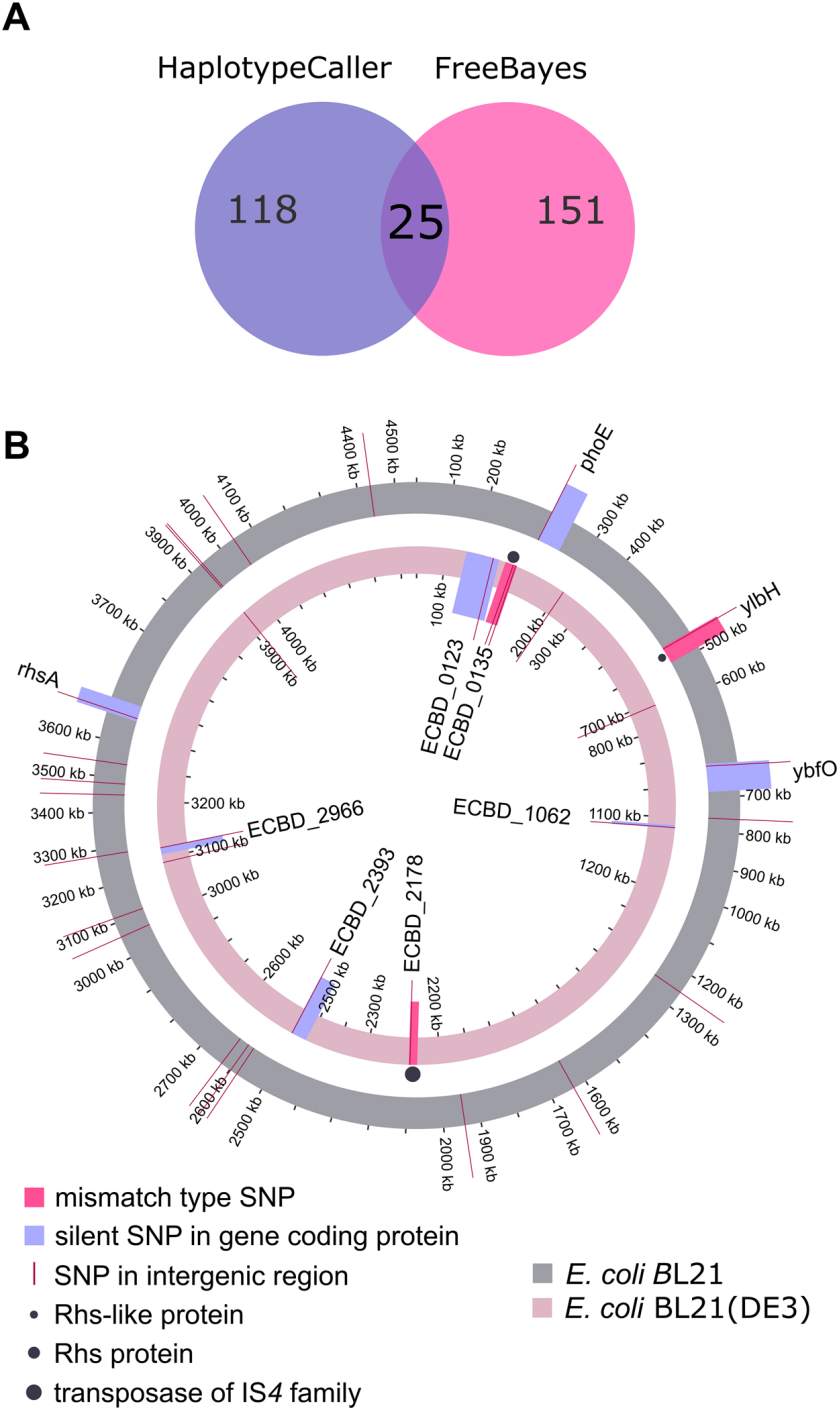
(**A**) Venn diagram for *E*. *coli* Bl21 showing SNPs detected by HaplotypeCaller and FreeBayes. (**B**) SNP causing mutations in genomes of *E*. *coli* BL21 and BL21(DE3) identified after 72 hours (three treatments) of exposition to ZnO nanorods. The outer ring (grey) shows mutations identified in the genome of *E*. *coli* BL21 and the inner one (pink) represents SNP mutations for *E*. *coli* BL21(DE3) genome. The scale is presented in kilobases (kb) showing the genome position. SNPs located in intergenic regions are marked with straight red lines, dark-pink areas indicate protein-coding sequences (genes) with mismatch type SNPs and violet areas represent genes with silent mutations. The length of protein-coding sequences is depicted by dark-pink and violet areas of different sizes. In the case of *E*. *coli* BL21 in the region between 1200 and 1300 kb, there are four SNPs marked as one line due to their close location. There is a similar situation in the region between 3100 and 3200 kb in *E*. *coli* BL21 where two SNPs were identified in close proximity. In the genome of *E*. *coli* BL21(DE3) in the region between 700 kb and 800 kb there are three SNPs marked as one line, and similarly in the regions between 3000 and 3100 kb and 3900 and 4000 kb, where there are two SNPs indicated by a red single line. Locus tags and/or names of genes with SNPs (both, silent and mismatch type) are indicated. The names of gene products are marked with dots.

We performed whole-genome sequencing for *E*. *coli* BL21 and *E*. *coli* BL21(DE3) exposed to the nanorods to examine changes in the genome. The control bacteria and the cells after exposure to sharp nanoparticles (3 experimental runs, 72 hours in total) were investigated. The DNA sequencing data for *E*. *coli* were analyzed by two programs: HaplotypeCaller and FreeBayes (see Fig. 4A). We decided to consider only point mutations detected by both programs for two strains. Figure 4B shows mutations identified in two bacterial strains that occurred in all three biological repeats of the experiment (details for SNPs identified for *E*. *coli* BL21 and *E*. *coli* BL21DE3 are provided in Supplementary Tables S1 and S2). Twenty-five single nucleotide polymorphisms (SNPs) were found in *E*. *coli* BL21. Interestingly, only one mismatch type SNP changed the protein coding sequence, while others were located within the intergenic regions or were silent mutations. In the case of *E*. *coli* BL21(DE3), sixteen SNPs were identified, and two SNPs were located within the protein-coding sequences. In both strains (each strain was obtained from a different, independent source) the SNP appeared in the region coding the Rhs protein or Rhs-like protein. The role of this gene is not clearly understood yet, however, it is suggested that the Rhs protein possesses binding properties and its function is associated with the cell surface^34,35^. In the case of *E*. *coli* BL21(DE3), one additional SNP was identified within the gene encoding the transposase of the IS*4* family, i.e. an enzyme responsible for transposition (transfer of mobile genetic elements). This mechanism is responsible for genome flexibility and the bacterial evolutionary process^36^. Although the majority of identified SNPs was localized within intergenic regions it is still possible that they are present within the not yet identified regions encoding ncRNAs, that may play important regulatory functions. Therefore, at least some of those SNPs may have a meaningful influence on the overall gene expression level.

### Transcriptome analysis

RNA sequencing was used to study the genes expression profiles of the bacteria after three exposures to ZnO nanorods (72 hours in total). Most of the experiments presented in this publication were performed on the bacteria harvested at the end of the logarithmic phase or in the stationary phase. To be consistent, we collected *E*. *coli* at the stationary phase for RNA-seq experiment. The same samples were used in whole transcriptome analysis and in DNA sequencing (see Fig. 4B).

RNA-seq analysis was fulfilled by 606 genes: 363 genes were up-regulated and 243 genes were down-regulated (see Supplementary Figs S10–15 in the Supplementary Information). Due to the fact that bacteria were harvested in the stationary phase the number of transcripts was relatively low, thus only genes with a high baseMean (the average number of reads mapped to a given gene in the control and in treated samples) and statistically significant padj <0.05 criterion (modified p-value corrected by multiple testing of the differences in gene expression using the Benjamini-Hochberg procedure^37^ were taken into consideration (more data provided in Table S3). These two criteria were fulfilled by 61 genes, all of which were down-regulated.

There were only 4 upregulated genes that fulfilled the statistical significance conditions (p-value < 1e-5, padj <0,002). All of them were 4–5 times upregulated on average: (i) WP_000502388.1: encoding putative selenate reductase subunit YgfK, (ii) WP_000953449.1: encoding TonB-dependent siderophore receptor that is the outer membrane receptor for ferric coprogen and ferric-rhodotorulic acid, involved in inorganic ion transport and metabolism, (iii) WP_000177906.1: encoding beta-galactosidase, (iv) WP_001026900.1: encoding a hypothetical protein, glycoside hydrolase family 127 protein.

The analysis revealed a lower expression of genes related to oxidation/degradation of fatty acids (eight genes in total). A lower expression of these genes may lead to a lower rate of β-oxidative cleavage of fatty acids into acetyl-CoA^38^ and may: (1) decrease the metabolism of the cell and (2) have an influence on the structure of the cell membrane that is composed of lipids^39^. The latter is supported by an observed higher minimum inhibitory concentration of colistin that acts as a surface active agent (see Fig. 3C). Different lipid composition of the cell membrane, being the result of decreased degradation of fatty acid, may lead to lower susceptibility to colistin.

A phenomenon of inhibited (slowed-down) general metabolism of the cells after exposure to the nanorods is reflected also by down-regulation of genes (ten genes in total) related to the tricarboxylic acid cycle (TCA) or genes that contribute to linking the glycolysis metabolic pathway to the citric acid cycle (three genes). This is because lipid metabolism results in the production of acetyl-CoA which is subsequently used in the TCA cycle. Down-regulation of the genes involved in: catabolism of galactitol, products of which are converted into intermediates of glycolysis (three genes), the glyoxylate cycle (a carbon metabolic pathway that is an alternative to the TCA cycle; two genes), and the glycolysis process (three genes) support the observation of metabolism inhibition upon exposure of bacteria to nanorods.

A lower expression of three genes encoding subunits of cytochrome bo(3) ubiquinol oxidase was observed. Cytochrome bo(3) ubiquinol oxidase is a terminal oxidase in the aerobic respiratory chain of *E*. *coli*, located in the cytoplasmic membrane^40^. This enzyme also possesses proton pump activity and translocates protons across the membrane, that results in building a potential gradient^41^. One of the key cofactors for this enzyme is iron, for which a lower amount was found in bacteria after exposure to the nanorods (determined by EDS, see Fig. 3B).

Interestingly, lower expression of three other genes, involved in uptake/conversion of iron was observed. This leads to a lower accumulation of the metal inside the cell. Consequently, this may affect many enzymes using iron as a cofactor e.g. cytochromes (proteins marked in the Supplementary Table S3 in the Supplementary Information).

Moreover, down-regulated expression was also observed for genes involved in: (1) transport of amino acids (two genes), (2) metabolism of amino acids (two genes), (3) cell division (two genes) and (4) peptide recycling during carbon starvation (two genes).

## Discussion

There are several publications on the influence of ZnO nanoparticles, which aim to evaluate cytotoxicity and dependence between the size of nanostructures and bactericidal effect^27,42–47^. Usually, the size of nanoparticles used in such experiments was in the order of a few nanometers up to dozens of nanometers, and the studied concentrations were in the range of µg per milliliter. The summary of the cytotoxic effect of ZnO nanostructures is given in our previous paper, where we studied the effect of the shape of particles on the viability of living cells^27^.

In this paper, we present data on phenotype plasticity of *E*. *coli* as a result of mechanical stress induced by sharp ZnO nanorods (size exceeding 300 nm and concentration of 1 mg ml^−1^).

Phenotype examination revealed that *E*. *coli*, which survived mechanical treatment changed the shape to more spherical. With the increased number of experimental runs and exposures to the stressor, the fraction of spherical cells increased. Jain *et al*. also noticed the change of shape of *E*. *coli* cells after exposure to ZnO nanorods. The authors concluded that the reshaping of bacteria can be a sign of distress since the cells were not able to divide^42^. The changes in the shape of *E*. *coli* upon exposure to mechanical stress that we observed could be the result of entering the viable but non-culturable (VBNC) state. This is a survival strategy adopted by bacteria exposed to different types of environmental stress (low temperature, starvation, ionic strength, irradiation, etc.). The characteristic feature of *E*. *coli* cells entering the VBNC state is a coccus-like shape^48^. However, there are three main differences between the state obtained through the mechanical stress and the VBNC state. First of all, bacteria in the VBNC state lose their cultivability in the media in which they normally grow so it is not possible to recover the cells when they are plated onto conventional growth media. In our case, the bacteria exposed to ZnO NR grew normally on lysogeny broth (LB-Miller medium), hence it was possible to determine growth curves by counting colony forming units (CFU) on agar plates. Secondly, the VBNC state is a result of changes only on a metabolic, proteome and gene expression level^48,49^. In contrast, we discovered 25 SNPs mutations in genomes of *E*. *coli* cells exposed to nanorods after DNA-seq analysis. And finally, since the bacteria in VBNC state have dramatically lowered metabolic activity^49^, they are not a target for most bacteriophages that prefer cells at a higher metabolic level^50^. We tested nanorods-treated cells if they are susceptible to bacteriophages and we obtained results similar to native (non-treated) bacteria. Plaques of phages (T1, P1, T4D, T4*rII*, TR4*rIII*, T7, λ) were properly formed without statistical differences in the efficiency of plating (see section 9 in the Supplementary Information and Supplementary Fig. S16). Statistically significant changes in growth kinetics of T4 bacteriophage in *E*. *coli* exposed to ZnO nanorods in comparison to control bacteria were also not observed. We measured the induction of the SOS response (induction of prophage), to check the level of DNA damages in *E*. *coli* exposed to ZnO nanorods, that is reflected in the number of released progeny phages. This experiment proved that DNA damages were not significant. More data for growth kinetics and induction of the SOS signal experiments are provided in the Supplementary Information in section 10 and 11 (Supplementary Figs S17 and S18 in the Supplementary Information), respectively.

Besides changes in size, we observed also increased thickness of the cell wall that additionally had higher density. Analysis of the elemental composition of the periplasm and the interior of *E*. *coli* performed by means of the energy dispersive X-ray spectroscopy revealed clear changes in the elemental composition upon exposure to external stress (Fig. 3B). Performed Gram staining of *E*. *coli* after exposure to the mechanical stress revealed a higher amount of peptidoglycan within the cell wall in comparison to control *E*. *coli*. (Fig. 1). Therefore, we tested whether a thicker cell wall could render bacteria more resistant to some specific antibiotics, especially those interfering with proteins involved in the cell envelope synthesis^51^. We found that the most pronounced changes in susceptibility between exposed and control *E*. *coli* were observed in the case of antibiotics interfering with the synthesis of peptidoglycan (Fig. 3C): (i) ampicillin that affects the cell cycle-related formation of peptidoglycan^52^; (ii) bacitracin which disrupts the transport of building blocks of the peptidoglycan bacterial cell wall outside the inner membrane (is not used to treat infection caused by Gram-negative bacteria)^53^, and (iii) cefazolin that inactivates penicillin binding proteins and as a result interferes with the cross-linkage of peptidoglycan chains necessary for bacterial cell wall integrity and rigidity^54,55^. What is meaningful, we observed down-regulation of genes involved in β-oxidation of the fatty acids, which may result in changes of the membranes structure, fluidity and consequently cell shape. This statement can be supported by an observed higher minimum inhibitory concentration for colistin^56,57^ that acts as a surface active agent (see Fig. 3C). Different lipid composition of the cell membrane, being the result of decreased degradation of fatty acid, may lead to lower susceptibility to colistin in comparison to control *E*. *coli* and control *S*. *epidermidis* (Gram-positive). Based on the results of Gram staining and TEM measurements (thickness of the cell wall), we suspected a difference in the stiffness of the cell wall of the bacteria after the exposure to ZnO nanorods. We used atomic force microscopy (AFM) to perform preliminary measurements of the Young modulus of the cell wall of the control *E*. *coli* cells and the bacteria after exposure to ZnO nanorods (data not shown). It can be concluded that the cell wall of the bacteria after exposure to ZnO nanorods is stiffer due to the fact that peptidoglycan is the component that contributes to the stiffness of the cell envelope in the greatest extent^58^. To conclude, all the presented results support the statement that *E*. *coli* after exposure to sharp ZnO nanorods possess thicker cell wall due to the higher amount of peptidoglycan.

DNA sequencing, performed on the whole population of *E. coli*, revealed a high number of SNPs detected by HaplotypeCaller and FreeBayes. However, we considered mutations identified by both programs. The reason for such pronounced differences in the number of detected SNP can be explained by the fact that physical stress induced by nanorods, as a selection factor, is not as strong as antibiotic treatment, where a specific mutation in particular locus is required for survival. Presumably, bacteria developed different mutations that could be spread in different loci in the genome. Depending on applied programs, that use different algorithms for SNP calling, some variations in detected mutations could occur, especially when we investigated the whole population with a high number of cells and different mutations. In this case, DNA sequencing on a single cell level would be more recommended and might reveal cellular heterogeneity on the genome level. It would be also relevant to perform follow up experiments to test whether the SNPs identified are important in the context of the nanorods treatment and the observed changes in phenotype.

Screening of the function of down-regulated genes revealed that they were mainly involved in metabolic pathways such as β-oxidation of fatty acids, glycolysis and the citric acid cycle, transport of amino acids, enzyme cofactors (e.g. iron) and other compounds used as sources of energy and “building blocks” for bacteria (more details in Supplementary Table S3). Based on obtained results we concluded that the overall rate of cellular metabolism is decreased by partial blocking of key metabolic pathways and down-regulation of energy production (e.g. by inhibiting glycolysis and transport of crucial substrates), and bacteria seem to invest energy and substrates in building stronger barriers, like thicker cell wall, isolating them from the environment (i.e. mechanical stressors).

In conclusion, our results show adaptation that is observable in a time scale comparable to the time of acquisition of resistance to antibiotics, i.e. hours^4,9^. The effect of exposure to physical contact with nanorods, in a turbulent flow in stirred medium, was fixed in phenotype and genome even when the stressing factor was removed. This was confirmed by TEM and EDS measurements performed after 16 hours, during which a small portion of the survivor bacteria was cultivated in the nanorods-free medium. We showed that the observed phenotypic plasticity of *E*. *coli* is a multilevel effect - a combination of changes on genome and transcriptome level. Thus, we have demonstrated the adaptation of bacteria to physical stress in a homogeneous environment with stable over time changes in phenotype, even without the presence of the mechanical stress.

## Materials and Methods

### Preparation of ZnO nanorods (NR)

ZnO nanostructures were synthesized using zinc nitrate and urotropine according to the aqueous chemical growth (ACG) method. After one hour of synthesis, the nanostructures were centrifuged, flushed twice with MiliQ (MQ) water and sonicated for 6 minutes. Sonication was applied to disrupt the sea urchin structures of ZnO and to obtain single, separated needles. The nanorods were flushed with MQ water and dried on a glass Petri dish (35 °C, 48 hours). The size of the nanorods was evaluated by scanning electron microscopy (SEM) after each synthesis. The nanorods were sterilized in a steam autoclave (Varioclav 3000 EP-Z, HP Medizintechnik, Germany) and suspended in growth medium just before adding to the cell culture. A detailed characterization of the NR obtained according to this method is provided elsewhere^27^.

### The procedure of bacteria exposure to ZnO NR

We used *E*. *coli* BL21 (obtained from the Faculty of Biology, University of Warsaw, Poland), *E*. *coli* BL21(DE3) (obtained from the Institute of Biochemistry and Biophysics of the Polish Academy of Sciences in Warsaw, Poland). The *E*. *coli* BL21(DE3) strain with two additional plasmids was resistant to chloramphenicol and kanamycin. First, a single colony from an agar plate was inoculated into LB medium (Roth) for seven hours (37 °C, 200 rpm). In the case of *E*. *coli* BL21(DE3) the medium was supplemented with chloramphenicol and kanamycin (Sigma-Aldrich) to final concentrations of 50 μg ml^−1^ and 25 µg ml^−1^, respectively. The overnight culture was inoculated into a new portion of LB medium (volume ratio 1:100) and cultured to obtain suspensions of OD_600_ = 0.1. The bacterial suspension was placed in two sterile flasks. The same starting amount of the bacteria was maintained. ZnO NR were added to one of the flasks to obtain a concentration of 1 mg ml^−1^. Bacteria were cultured in a shaker (IKA KS 4000 i, Germany) for 24 hours (37 °C, 200 rpm). In the case of a higher number of exposures, after 24 hours, 100 µl of control bacteria were inoculated into a fresh portion of LB medium, and similarly 100 µl of the cells that had survived the first exposure to sharp ZnO nanorods (NR) were inoculated into the fresh portion of LB medium supplemented with the nanorods. Bacteria were cultured for 24 hours in the shaker. To determine the viability of the bacteria upon exposure to ZnO nanorods (NR), we applied the colony count method (at least 6 technical replicates for each dilution) to 50 µl of the bacterial suspension was seeded per agar plate. After 24 hours of incubation on Petri dishes, the bacteria were counted to determine the colony forming units (CFU) in one millilitre. The whole experiment was performed in three independent repeats.

### UV spectra of Gram-stained bacteria

Gram staining was performed for the *E*. *coli* BL21(DE3) strain after two exposures to ZnO nanorods (each exposure for 24 hours). The bacteria were left for 5 minutes motionless in order to decrease the number of nanorods by sedimentation and the supernatant was further analyzed. Samples of the bacteria were prepared by adjusting to the same OD ~ 1.0. Subsequently, the bacteria were centrifuged (5 minutes, 4000 rpm) and the pellet was suspended in sterile physiological saline (0.9% mass/volume). The sodium chloride needed to prepare the physiological saline was purchased from Sigma-Aldrich. A smear of bacterial suspension was made on a clean glass slide by pipetting 250 µl of suspension, left to dry in the air and heat fixed by passing it several times through a flame. The sample was flooded with crystal violet for 30 seconds and washed with sterile MQ water. Then the glass slide was treated with Lugol solution for 60 seconds and rinsed with water. Ethanol (96%, POCH, Poland) was poured over the smear for about 3–4 seconds and the samples were washed with a large volume of water. The glass slide was flooded with safranine for 60 seconds, rinsed with water and drained. The crystal violet, Lugol solution, and safranine were purchased from Aqua-Med Company (Poland). Images of the stained bacteria were taken using the Nikon Eclipse 50i (Japan) microscope. The smears from the plates (control and upon exposure to NR) were entirely washed using 4 ml of a mixture of ethanol and water (50% volume/volume). UV-Vis spectra were recorded using the UV-Visible Spectrophotometer Evolution 201 (Thermo Scientific, USA).

### Scanning electron microscopy (SEM) and cryo-scanning electron microscopy (Cryo-SEM)

Cell shapes of *E*. *coli* BL21 and *E*. *coli* BL21(DE3) were observed using a SEM microscope. In the case of SEM imaging, a special protocol for bacterial preparation was established to avoid several centrifugation steps that are required during the exchange of solvents. In samples where the bacteria were suspended with the nanorods, the centrifugal force could intensify the piercing of the bacteria by the nanorods that would falsify the real pictures of the cells. The bacterial cells *E*. *coli* BL21 (control and after exposure to NR every 24 hours) were centrifuged (4 minutes, 4000 rpm). The bacterial pellet was suspended in a sterile, 50-fold diluted solution of physiological saline in MQ water. A suspension of the bacteria (5 µl) was placed on (previously washed in acetone, ethanol, MQ water) silicon plates. After 60 seconds half the liquid was discarded to obtain a thin film on the surface of the plate. Just as the edges of the spot were starting to dry out, the plates were gently flushed by a rowing movement in filtered MQ water to remove the excess salt from the surface. Silicon plates were attached to the metal holder by silver paste. Images were taken using the FEI Nova NanoSEM 450 (USA) scanning electron microscope. In the case of cryo-SEM, *E*. *coli* BL21(DE3) cells after two exposures to NR (each exposure for 24 hours) were analyzed. Images were taken with the use of the JEOL JSM-7001F TTLS (JEOL Ltd., Japan) scanning electron microscope equipped with the PP3000T cryo-SEM preparation system which enables the preparation, loading, processing, and transfer of cryo specimens into the SEM chamber. The sample was prepared by dropping 50 µl of bacterial suspension on the metal holder. The sample was rapidly frozen by plunging the holder into slush nitrogen (the temperature around −210 °C) and transferred under a vacuum to the preparation chamber mounted onto the SEM. Inside the preparation chamber (−185 °C), the specimen was fractured to expose a fresh surface. Then it was sublimated and coated with a thin platinum layer. Finally, the sample was transferred under a vacuum into the SEM cryo stage (−190 °C) where the surface was imaged applying an accelerating voltage of 10 kV and secondary electron (SEI) detector.

### Transmission electron microscopy (TEM) and X-ray microanalysis

*E*. *coli* BL21(DE3) cell wall thickness was measured after 24-hours exposure to the nanorods. 100 µl of the bacteria from the control and the nanorod-treated culture was inoculated into a fresh portion of LB medium in order to remove the nanoparticles. Overnight cultures were centrifuged (5 minutes, 4000 rpm) and suspended in 0.9% physiological saline. The bacteria were fixed in 1.5% glutaraldehyde (final concentration) in 0.1 M cacodylate buffer (pH = 7.2) for 2 hours with mixing (Biosan Mini rocker MR-1; max speed) at room temperature and next washed twice in the same buffer followed by gentle centrifugation after each step. The cultures were divided into two parts: one part was post-fixed in 2% osmium tetroxide for 1 h (with gentle shaking), while the other was not. After washing in cacodylate buffer the samples were dehydrated (50% and 70% ethanol), centrifuged and left O/N in 70% ethanol. After dehydration in a graded series of ethanol solutions followed by 3 changes (30 minutes each) of the mixture of LR-White resin (Polysciences, Inc., Warrington, PA) with 100% ethanol (at 1:1, 2:1, and 3:1 ratio, respectively) on a rotator, the samples were infiltrated with LR-White O/N, and embedded in this resin. After thin-sectioning, samples were collected on copper or nickel grids, respectively (Agar Scientific Ltd., Stansted, UK). Ultrastructure and X-ray microanalysis of the specimens was carried out with the transmission electron microscope JEM 1400 (JEOL Co., Japan), equipped with a digital camera (CCD MORADA, SiS-Olympus, Germany) and an energy-dispersive full range X-ray microanalysis system EDS INCA (The Microanalysis Suite, Issue 18, ver. 4.11, Energy TEM 250, Oxford Instruments, UK) in an energy range of 1–10 kV at an accelerating voltage of 80 kV (Livetime 300.0 s) in the Laboratory of Electron Microscopy (Nencki Institute of Experimental Biology, PAS). For thickness measurements (TEM) the following number of cells was measured: (1) nickel meshes: 29 control cells and 25 cells after exposure to ZnO nanorods (NR); (2) nickel meshes with osmium tetroxide saturation: 58 control cell and 67 cells after exposure to NR; (3) copper meshes: 36 control cells and 36 cells after exposure to NR; (4) copper meshes with osmium tetroxide saturation: 19 control cells and 43 cells after exposure to NR. In the case of X-ray microanalysis the analysis of the cytoplasm and the periplasm was performed using the following numbers of bacteria: (1) nickel meshes: 22 control cells and 22 cells after exposure to ZnO nanorods (NR); (2) nickel meshes with osmium tetroxide saturation: 11 control cell and 8 cells after exposure to NR; (3) copper meshes and copper meshes with osmium tetroxide saturation: 12 control cells and 12 cells after exposure to NR.

### Minimum inhibitory concentration (MIC) evaluation

The MIC was evaluated using the E-test with a predefined antibiotic gradient immobilized on one side of the plastic strip. E-tests with ampicillin, bacitracin, cefazolin, colistin, kanamycin, tetracycline (Biomaxima, Poland) were used in the studies of *E*. *coli* BL21 (Gram-negative strain): control cells and upon exposure to the nanorods. *S*. *epidermidis* was used as the referential Gram-positive strain. E-tests for all antimicrobials were used in a gradient of antibiotic concentrations of 0.016–256 µg ml^−1^. First, the bacteria were exposed to ZnO nanorods (NR) (described above). *S*. *epidermidis* was used as a reference Gram-positive strain. 250 µl of each bacterial suspension after first exposure (control *E*. *coli*; *E*. *coli* after exposure to NR, control *S*. *epidermidis*) was inoculated into a new portion of LB medium (25 ml) or LB medium with suspended ZnO NR at a concentration of 1 mg ml^−1^ (in the case of exposed *E*. *coli*). After the second exposure, the flask with ZnO NR was left for 5 minutes to enable sedimentation of the larger aggregates. Next, the suspensions of the bacteria after the experiment were used to prepare inoculum in 0.9% physiological saline, where the turbidity was adjusted to 0.5 McFarland standard. Standardized Mueller Hinton (MH) agar broth (Biocorp, Poland) was used to assess the MIC concentration. Bacterial suspensions were spread on dry MH agar plates using sterile swabs. E-tests were put on agar plates with the bacteria and after 20 hours of incubation, the MIC values were read. Two biological repeats of the experiment were performed, for each type of the sample two technical replicates were done.

### Statistical analysis

Significant differences between control *E*. *coli* and *E*. *coli* upon exposure to ZnO nanorods in the case of SEM, cryo-SEM, TEM, EDS, and antibiotic susceptibility data were determined using the unpaired t-test.

### Isolation of bacterial DNA and genome sequencing

Genome studies were performed for *E*. *coli* BL21 and *E*. *coli* BL21(DE3). Bacteria were exposed to the nanorods three times (for 24 h run each). DNA isolation was performed using a commercial GeneMATRIX Bacterial & Yeast Genomic DNA Purification Kit (EURx, Poland), according to the protocol for Gram-negative bacteria. Evaluation of quantity and quality of DNA was done spectrophotometrically using NanoDrop (Thermo Fisher Scientific, USA). The quantity of isolated material was measured fluorometrically on Qubit (Life Technologies, USA) using the dsDNA HS Assay (Life Technologies, USA) reagent kit. Library preparation was performed on diluted DNA (0.2 ng µl^−1^) with the Nextera XT DNA Sample Preparation Kit (Illumina, USA). The quality and quantity were evaluated by chip electrophoresis on the 2100 Bioanalyzer (Agilent, USA) using the Agilent High Sensitivity DNA Kit (Agilent, USA). Additionally, the quantity was checked fluorometrically on Qubit. PE 2 × 150 DNA sequencing was performed on the MiSeq device (Illumina, USA).

### Genome analysis and SNP discovery

At first, DNA reads were searched for contaminations and sequencing adapters using the FastQ screen with standard settings. Program Blue was used for error correction. The Flexbar was utilized to remove adapters, low-quality reads, over-represented sequences, and short sequences. Purified reads were used to perform mapping, for each sample separately, using the Bowtie2 aligner. The best sequence for *E*. *coli* BL21 was stored in the GeneBank with reference number AM946981 and referred as *E*. *coli* BL21(DE3) ASM956v1. For *E*. *coli* BL21(DE3) the best reference sequence was referred to BL21-Gold(DE3)pLysS AG in GeneBank. Variants were discovered using the FreeBayes and Haplotype Caller with standard settings for each sample separately. For each strain and sample type, three biological replicates were prepared. All groups were filtered out to contain variants which were common to all three biological replicates and were discovered by both the FreeBayes and Haplotype Caller. VCFtools was used to compare results for all groups. Annotation of the discovered variants was executed with ANNOVAR.

### Isolation of bacterial RNA

*E*. *coli* BL21 bacteria were exposed to nanorods three times in 24-hour runs in order to enrich the sample with more spherical cells (according to the procedure described above). The total RNA was isolated from the bacteria using the commercial GeneMatrix Universal RNA purification kit (EURx. Poland). The quantity was evaluated spectrophotometrically using Nanodrop (Thermo Fisher Scientific. USA) and fluorometrically on Qubit (Life Technologies, theUSA) using the Qubit RNA HS Assay kit (Thermo Fisher Scientific, the USA). The quality of isolated RNA was additionally checked by electrophoresis in 1% agarose gel. Depletion of rRNA in samples was performed using the Ribominus^TM^ Transcriptome Isolation Kit (Invitrogen, USA) according to the instructions attached to the kit. Double-stranded DNA was obtained using the Maxima H Minus Double-stranded cDNA Synthesis Kit (Thermo Fisher Scientific, USA). The PCR Purification Kit (Qiagen, Netherlands) was used to purify cDNA and its quantity was then measured fluorometrically on Qubit. Library preparation was conducted using the Nextera XT DNA Sample Preparation Kit (Illumina, USA). The quality and quantity of the obtained libraries were evaluated on the 2100 Bioanalyzer (Agilent, the USA) using the Agilent High Sensitivity DNA Kit (Agilent. USA) and Qubit. Sequencing of DNA was performed on the MiSeq device (Illumina, USA; v2 kit PE 2 × 150 cycles).

### The procedure of gene expression analysis

The quality of raw RNA-Seq datasets were evaluated using FastQC and then processed with Trimmomatic v0.36 to remove adaptor sequences and low-quality nucleotides using the following command ILLUMINACLIP:ADAPTORS:2:30:10 LEADING:5 TRAILING:5 SLIDINGWINDOW:15:20 MINLEN:36^59^. The resulting reads for each replicate were mapped against *E*. *coli* BL21(DE3) genome (Accession Number: NC_012892.2) with HISAT2 v2.1.0 in unstranded mode using separately paired and unpaired reads^60^. Counts of reads mapping to each gene were performed with htseq-count v.0.9.0 on paired and single reads in union mode and combined afterward to reflect the total count for each sequencing sample^61^. The analysis of differentially expressed genes was conducted based on acquired count tables and analyzed with DESeq2 v1.16.1^62^. Figures S10–S15 represent rlog transformed count tables of significantly differentially expressed genes (padj <0.05) generated with ggplot2 v.2.2.1 (H. Wickham. ggplot2: Elegant Graphics for Data Analysis. Springer-Verlag New York, 2009).

## Supplementary information

Supplementary information

## Data Availability

All data generated or analyzed during this study are included in the manuscript and the Supplementary Information.

## References

[1] Lenski RE, Rose MR, Simpson SC, Tadler SC (1991). Long-Term Experimental Evolution in *Escherichia coli*. I. Adaptation and Divergence During 2,000 Generations. Am. Nat..

[2] Barrick JE (2009). Genome evolution and adaptation in a long-term experiment with *Escherichia coli*. Nature.

[3] Tenaillon O (2016). Tempo and mode of genome evolution in a 50,000-generation experiment. Nature.

[4] Baym, M. *et al*. Spatiotemporal microbial evolution on antibiotic landscapes. *Science* (*80-*). **353** (2016). 10.1126/science.aag0822 PMC5534434 27609891

[5] Brown D (2015). Antibiotic resistance breakers: can repurposed drugs fill the antibiotic discovery void?. Nat. Rev. Drug Discov..

[6] Brown ED, Wright GD (2016). Antibacterial drug discovery in the resistance era. Nature.

[7] Crofts TS, Gasparrini AJ, Dantas G (2017). Next-generation approaches to understand and combat the antibiotic resistome. Nat. Rev. Microbiol..

[8] Wright GD (2007). The antibiotic resistome: the nexus of chemical and genetic diversity. Nat. Rev. Microbiol..

[9] Zhang Q (2011). Acceleration of Emergence of Bacterial Antibiotic Resistance in Connected Microenvironments. Science (80-)..

[10] Pillai PP, Kowalczyk B, Kandere-Grzybowska K, Borkowska M, Grzybowski BA (2016). Engineering Gram Selectivity of Mixed-Charge Gold Nanoparticles by Tuning the Balance of Surface Charges. Angew. Chemie.

[11] Wybrańska K (2015). Gold–Oxoborate Nanocomposites and Their Biomedical Applications. ACS Appl. Mater. Interfaces.

[12] Lam SJ (2016). Combating multidrug-resistant Gram-negative bacteria with structurally nanoengineered antimicrobial peptide polymers. Nat. Microbiol..

[13] Rai MK, Deshmukh SD, Ingle AP, Gade AK (2012). Silver nanoparticles: the powerful nanoweapon against multidrug-resistant bacteria. J. Appl. Microbiol..

[14] Haider A, Kang I-K, Haider A, Kang I-K (2015). Preparation of Silver Nanoparticles and Their Industrial and Biomedical Applications: A Comprehensive Review, Preparation of Silver Nanoparticles and Their Industrial and Biomedical Applications: A Comprehensive Review. Adv. Mater. Sci. Eng. Adv. Mater. Sci. Eng..

[15] Rauwel P (2015). Silver Nanoparticles: Synthesis, Properties, and Applications, Silver Nanoparticles: Synthesis, Properties, and Applications. Adv. Mater. Sci. Eng. Adv. Mater. Sci. Eng..

[16] de Lima R, Seabra AB, Durán N (2012). Silver nanoparticles: a brief review of cytotoxicity and genotoxicity of chemically and biogenically synthesized nanoparticles. J. Appl. Toxicol. JAT.

[17] Gupta A, Silver S (1998). Silver as a biocide: will resistance become a problem?. Nat. Biotechnol..

[18] Li XZ, Nikaido H, Williams KE (1997). Silver-resistant mutants of *Escherichia coli* display active efflux of Ag+ and are deficient in porins. J. Bacteriol..

[19] Hendry AT, Stewart IO (1979). Silver-resistant Enterobacteriaceae from hospital patients. Can. J. Microbiol..

[20] Hajipour MJ (2012). Antibacterial properties of nanoparticles. Trends Biotechnol..

[21] Stark WJ (2011). Nanoparticles in Biological Systems. Angew. Chemie Int. Ed..

[22] Cady NC, Behnke JL, Strickland AD (2011). Copper-Based Nanostructured Coatings on Natural Cellulose: Nanocomposites Exhibiting Rapid and Efficient Inhibition of a Multi-Drug Resistant Wound Pathogen, A. baumannii, and Mammalian Cell Biocompatibility *In Vitro*. Adv. Funct. Mater..

[23] Lichter JA, Rubner MF (2009). Polyelectrolyte Multilayers with Intrinsic Antimicrobial Functionality: The Importance of Mobile Polycations. Langmuir.

[24] Ivanova EP (2012). Natural bactericidal surfaces: mechanical rupture of Pseudomonas aeruginosa cells by cicada wings. Small.

[25] Pogodin S (2013). Biophysical Model of Bacterial Cell Interactions with Nanopatterned Cicada Wing Surfaces. Biophys. J..

[26] Tripathy A, Sen P, Su B, Briscoe WH (2017). Natural and bioinspired nanostructured bactericidal surfaces. Adv. Colloid Interface Sci..

[27] Matuła K (2016). Influence of nanomechanical stress induced by ZnO nanoparticles of different shapes on the viability of cells. Soft Matter.

[28] Raghupathi KR, Koodali RT, Manna AC (2011). Size-dependent bacterial growth inhibition and mechanism of antibacterial activity of zinc oxide nanoparticles. Langmuir ACS J. surfaces colloids.

[29] Nel A, Xia T, Mädler L, Li N (2006). Toxic potential of materials at the nanolevel. Science.

[30] Sneddon IN (1965). The relation between load and penetration in the axisymmetric boussinesq problem for a punch of arbitrary profile. Int. J. Eng. Sci..

[31] Cayley DS, Guttman HJ, Record MT (2000). Biophysical characterization of changes in amounts and activity of *Escherichia coli* cell and compartment water and turgor pressure in response to osmotic stress. Biophys. J..

[32] Lan G, Wolgemuth CW, Sun SX (2007). Z-ring force and cell shape during division in rod-like bacteria. Proc. Natl. Acad. Sci. USA.

[33] Liu S (2010). Antibacterial action of dispersed single-walled carbon nanotubes on *Escherichia coli* and Bacillus subtilis investigated by atomic force microscopy. Nanoscale.

[34] Hill CW, Sandt CH, Vlazny DA (1994). Rhs elements of *Escherichia coli*: a family of genetic composites each encoding a large mosaic protein. Mol. Microbiol..

[35] Wang YD, Zhao S, Hill CW (1998). Rhs elements comprise three subfamilies which diverged prior to acquisition by *Escherichia coli*. J. Bacteriol..

[36] De Palmenaer D, Siguier P, Mahillon J (2008). IS4 family goes genomic. BMC Evol. Biol..

[37] McDonald, J. H. *Handbook of biological statistics, Second edition* (2009).

[38] Fujita Y, Matsuoka H, Hirooka K (2007). Regulation of fatty acid metabolism in bacteria. Mol. Microbiol..

[39] Polyak SW, Abell AD, Wilce MCJ, Zhang L, Booker GW (2012). Structure, function and selective inhibition of bacterial acetyl-coa carboxylase. Appl. Microbiol. Biotechnol..

[40] Iwata S (2000). The structure of the ubiquinol oxidase from *Escherichia coli* and its ubiquinone binding site. Nat. Struct. Biol..

[41] Yap LL (2010). The quinone-binding sites of the cytochrome bo3 ubiquinol oxidase from *Escherichia coli*. Biochim. Biophys. Acta.

[42] Jain A, Bhargava R, Poddar P (2013). Probing interaction of Gram-positive and Gram-negative bacterial cells with ZnO nanorods. Mater. Sci. Eng. C.

[43] Reddy KM (2007). Selective toxicity of zinc oxide nanoparticles to prokaryotic and eukaryotic systems. Appl. Phys. Lett..

[44] Wahab R, Mishra A, Yun S-I, Kim Y-S, Shin H-S (2010). Antibacterial activity of ZnO nanoparticles prepared via non-hydrolytic solution route. Appl. Microbiol. Biotechnol..

[45] Stankic S, Suman S, Haque F, Vidic J (2016). Pure and multi metal oxide nanoparticles: synthesis, antibacterial and cytotoxic properties. J. Nanobiotechnology.

[46] Stanković A, Dimitrijević S, Uskoković D (2013). Influence of size scale and morphology on antibacterial properties of ZnO powders hydrothemally synthesized using different surface stabilizing agents. Colloids Surfaces B Biointerfaces.

[47] Ramani M, Ponnusamy S, Muthamizhchelvan C (2012). From zinc oxide nanoparticles to microflowers: A study of growth kinetics and biocidal activity. Mater. Sci. Eng. C.

[48] Signoretto C, Lleò MM, Canepari P (2002). Modification of the peptidoglycan of *Escherichia coli* in the viable but nonculturable state. Curr. Microbiol..

[49] Oliver JD (2005). The viable but nonculturable state in bacteria. J. Microbiol..

[50] Chibani-Chennoufi S, Bruttin A, Dillmann M-L, Brüssow H (2004). Phage-host interaction: an ecological perspective. J. Bacteriol..

[51] Kohanski MA, Dwyer DJ, Collins JJ (2010). How antibiotics kill bacteria: from targets to networks. Nat. Rev. Microbiol..

[52] Tipper DJ (1985). Mode of action of beta-lactam antibiotics. Pharmacol. Ther..

[53] Stone KJ, Strominger JL (1971). Mechanism of action of bacitracin: complexation with metal ion and C 55 -isoprenyl pyrophosphate. Proc. Natl. Acad. Sci. USA.

[54] Truesdell SE, Zurenko GE, Laborde AL (1989). Interaction of cephalosporins with penicillin-binding proteins of methicillin-resistant Staphylococcus aureus. J. Antimicrob. Chemother..

[55] Yotsuji A (1988). Mechanism of action of cephalosporins and resistance caused by decreased affinity for penicillin-binding proteins in Bacteroides fragilis. Antimicrob. Agents Chemother..

[56] Deane J., Rea M.C., Fouhy F., Stanton C., Ross R.P., Plant B.J. (2016). Long-Term Implications of Antibiotic Use on Gut Health and Microbiota in Populations Including Patients With Cystic Fibrosis. The Gut-Brain Axis.

[57] Bradley, J. S. & Sauberan, J. B. Antimicrobial Agents. *Princ*. *Pract*. *Pediatr*. *Infect*. *Dis*. 1453–1484.e5, 10.1016/B978-1-4377-2702-9.00294-4 (2012).

[58] Auer GK, Weibel DB (2017). Bacterial Cell Mechanics. Biochemistry.

[59] Bolger AM, Lohse M, Usadel B (2014). Trimmomatic: a flexible trimmer for Illumina sequence data. Bioinformatics.

[60] Pertea M, Kim D, Pertea GM, Leek JT, Salzberg SL (2016). Transcript-level expression analysis of RNA-seq experiments with HISAT, StringTie and Ballgown. Nat. Protoc..

[61] Anders S, Pyl PT, Huber W (2015). HTSeq–a Python framework to work with high-throughput sequencing data. Bioinformatics.

[62] Love MI, Huber W, Anders S (2014). Moderated estimation of fold change and dispersion for RNA-seq data with DESeq2. Genome Biol..

