# Supplementary material for: Phenotypic plasticity of Escherichia coli upon exposure to physical stress induced by ZnO nanorods
Source: Sci Rep. 2019 Jun 12;9:8575. doi: 10.1038/s41598-019-44727-w (PMC6561948; doi:10.1038/s41598-019-44727-w)
Supplement: Supplementary file 1 — Supplementary information [file 41598_2019_44727_MOESM1_ESM.pdf]

Supplementary information

Phenotypic plasticity of *Escherichia coli* upon exposure to physical stress induced by ZnO nanorods

Kinga Matuła[1], Łukasz Richter[1], Marta Janczuk-Richter[1], Wojciech Nogala[1], Mikołaj Grzeszkowiak[2], Barbara Peplińska[2], Stefan Jurga[2], Elżbieta Wyroba[3], Szymon Suski[3], Henryk Bilski[3], Adrian Silesian[4], Hans A. R. Bluyssen[4], Natalia Derebecka[5], Joanna Wesoły[5], Joanna M. Łoś[6,7], Marcin Łoś[6,7], Przemyslaw Decewicz[8], Lukasz Dziewit[8], Jan Paczesny[1], Robert Hołyst[1]

[1]Institute of Physical Chemistry of the Polish Academy of Sciences, Kasprzaka 44/52, 01-224 Warsaw, Poland

[2]Nanobiomedical Centre, Adam Mickiewicz University, Umultowska 85, 61-614, Poznań, Poland

[3]Nencki Institute of Experimental Biology of the Polish Academy of Sciences, 3 Pasteur Street, 02-093 Warsaw, Poland

[4]Department of Human Molecular Genetics, Institute of Biotechnology and Molecular Biology, Faculty of Biology, Adam Mickiewicz University, Umultowska 89, 61-614, Poznań, Poland

[5]Laboratory of High Throughput Technologies and Department of Human Molecular Genetics, Institute of Biotechnology and Molecular Biology, Faculty of Biology, Adam Mickiewicz University, Umultowska 89, 61-614, Poznań, Poland

[6]Department of Molecular Genetics of Bacteria, Faculty of Biology, University of Gdańsk, Wita Stwosza 59, 80-308 Gdańsk, Poland

[7]Phage Consultants, Partyzantów 10/18, 80-254 Gdańsk, Poland

[8]Department of Bacterial Genetics, Institute of Microbiology, Faculty of Biology, University of Warsaw, Miecznikowa 1, 02-096 Warsaw, Poland

Corresponding authors

Jan Paczesny, Fax: 48 22 343 3333; Tel: 48 22 3432071; E-mail: jpaczesny@ichf.edu.pl

Robert Hołyst, Fax: 48 22 343 3333; Tel: 48 22 3433407; E-mail: rholyst@ichf.edu.pl

Table of contents

1. Additional tables and Figures. 3

2. Hertzian theory of collisions: interaction between *Escherichia coli* bacteria cells and ZnO nanorods 13

3. Viability curves of *Escherichia coli* bacteria exposed to ZnO nanostructures 15

4. Dependence of the viability curves on the initial inoculum of *E. coli* 17

5. Supplementary comparison of UV-Vis spectra after Gram staining of *E. coli* exposed to ZnO nanorods and spherical ZnO nanoparticles 18

6. Change of shape of *Enterobacter aerogenes* (Gram-negative) after exposure to ZnO nanorods 19

7. Supplementary transmission electron images (TEM) of *Escherichia coli* bacteria exposed to ZnO nanorods 21

8. Examination of transcriptome by RNA sequencing. 22

9. Testing of phage susceptibility of *E. coli* exposed to ZnO nanorods 29

10. Growth kinetics of T4 bacteriophage in *E. coli* exposed to ZnO nanorods. 31

11. Induction of the SOS response 34

12. References 37

1. Additional tables and Figures.

Supplementary Table 1. Details for SNP mutations identified in *E. coli* BL21.

Escherichia coli BL21						
**Gene**	**Genome position**	**Reference**	**Variant**	**Type**	**Amino acid change**	**Description of gene product**
phoE	263558	C	A	synonymous variant	p.Ala344Ala	outer membrane pore protein E
ylbH	**497181**	**C**	**G**	**missense variant**	**p.His31Gln**	**conserved Rhs-like protein**
ybfO	691808	G	A	synonymous variant	p.Thr24Thr	conserved rhs-like protein
Rz/borD	766400	A	C	intergenic region	-	DLP12 prophage; predicted murein endopeptidase/bacteriophage lambda Bor protein homologue
rdlD/ldrB	1257654	G	A	intergenic region	-	RdlD antisense regulatory RNA of the LdrD-RdlD toxin-antitoxin system/ small toxic polypeptide LdrB
rdlD/ldrB	1257661	G	A	intergenic region	-	
rdlD/ldrB	1257670	T	C	intergenic region	-	
rdlD/ldrB	1257686	A	G	intergenic region	-	
ydgA/uidC	1636657	C	T	intergenic region	-	conserved protein with unknown function/membrane-associated protein
hchA_2	1941183	T	A	pseudogene	-	-
srmB/yfiE	2580938	C	T	intergenic region	-	DEAD-box RNA helicase/DNA-binding transcriptional regulator LYSR-type
rrsG/clpB	2597817	C	T	intergenic region	-	16S ribosomal RNA /ClpB chaperone
ybl116/insA-15	2627707	G	T	intergenic region	-	ybl116/IS1 protein InsA
yqiK/SIB_RNA	3060713	A	G	intergenic region	-	inner membrane protein YqiK/Sib RNA
yhaC/RNaseP_bact_a	3136047	C	A	intergenic region	-	uncharacterized protein YhaC/ribonuclease P
yhaC/RNaseP_bact_a	3136086	A	C	intergenic region	-	
yhdZ/rrfF	3284549	C	T	intergenic region	-	component of YhdW/YhdX/YhdY/YhdZ ABC transporter/5S ribosomal RNA
ugpB/livF	3454964	G	A	intergenic region	-	sn-glycerol-3-phosphate-binding periplasmic protein Ugp/subunit of leucine ABC transporter
yrhC/yhhI	3486576	T	C	intergenic region	-	Pseudogene/putative transposase

bcsG/ldrD	3561157	A	G	intergenic region	-	inner membrane protein/LdrD peptide of the LdrD-RdlD toxin-antitoxin system
rhsA	3628692	A	G	synonymous variant	p.Gly42Gly	RhsA protein in rhs element
fre/fadA	3934314	T	C	intergenic region	-	FMN reductase/ 3-ketoacyl-CoA thiolase
hemG/rrsA	3942070	G	C	intergenic region	-	protoporhyrinogen oxidase/16S ribosomal RNA
yijF/gldA	4046396	T	G	intergenic region	-	conserved protein with unknown function/D-aminopropanol dehydrogenase
insAB-26	4432523	A	C	pseudogene	-	-

Supplementary Table 2. Details for SNP mutations identified in *E. coli* BL21(DE3).

Escherichia coli BL21(DE3)						
**Gene**	**Genome position**	**Reference**	**Variant**	**Type**	**Amino acid change**	Description of gene product
ECBD_0123	129347	A	G	synonymous variant	p.Asn700An	YadA domain protein
ECBD_0135	142160	C	T	synonymous variant	p.Arg119Ag	**Rhs protein**
**ECBD_0135**	**142420**	**T**	**G**	**missense variant**	**p.Lys33Gln**	
ECBD_0200/ ECBD_0210	211766	G	T	intergenic region	-	hypothetical protein/cellulose synthase operon protein YhjU
ECBD_0689/ ECBD_0690	713101	T	G	intergenic region	-	D-beta-D-heptose 7-phosphate kinase, D-beta-D-heptose 1-phosphate adenosyltransferas/band 7 protein
	713111	T	C	intergenic region	-	
	713152	T	G	intergenic region	-	
ECBD_1062	1112063	A	G	synonymous variant	p.Thr24Thr	insertion element protein PFAM
**ECBD_2178**	**2293820**	**G**	**T**	**missense variant**	**p.Ala219Gu**	**transposase of IS*4* family**
ECBD_2393	2510574	G	A	synonymous variant	p.His20His	nitrate reductase subunit beta
ECBD_2958/ ECBD_2959	3090512	C	T	intergenic region	-	pseudogene/hypothetical protein
	3090571	C	T	intergenic region	-	
ECBD_2966	3101368	A	G	synonymous variant	p.Gly197Gly	DNA-binding transcriptional activator KdpE response regulator
ECBD_3744	3910203	A	C	synonymous variant	-	pseudogene
ECBD_3744/ ECBD_3745	3910560	T	C	intergenic region	-	pseudogene/RNA polymerase sigma factor FecI
	3910565	T	A	intergenic region	-	

Supplementary Table 3. Details for gene expression. Log2FoldChange is log2 from the difference in expression of the gene in the control sample in comparison with bacteria exposed to nanorods; gene product is the name of the protein encoded by the gene, function of which is shown in the last column in the table below. The functions of genes were found in UniProt and BioCyc databases. In the table genes with padj from 4.79E-18 to 0.04095 are shown.

**Gene**	**Log2 Fold Change**	**Gene product**	**The Function of the gene product**
**B21_RS03155 (gltL)**	-3.93	glutamate/aspartate ABC transporter substrate-binding protein	involved in glutamate and aspartate uptake
**B21_RS15760 (mtr)**	-2.46	tryptophan-specific transporter	involved in transporting tryptophan across the cytoplasmic membrane
**B21_RS01990 (cyoD)**	-4.44	cytochrome bo(3) ubiquinol oxidase subunit 4	the component of the aerobic respiratory chain that predominates when cells are grown at high aeration; has proton pump activity across the membrane
**B21_RS01995 (cyoC)**	-3.21	cytochrome bo(3) ubiquinol oxidase subunit 3	involved in the aerobic respiratory chain of *E. coli* that predominates when cells are grown at high aeration; has proton pump activity across the membrane in addition to electron transfer
**B21_RS02000 (cyoB)**	-2.27	cytochrome ubiquinol oxidase subunit 1	involved in aerobic respiratory chain that predominates when cells are grown at high aeration; has proton pump activity across the membrane
**B21_RS03510 (sdhA)**	-3.20	succinate dehydrogenase flavoprotein subunit	involved in step 1 of the subpathway that synthesizes fumarate from succinate
**B21_RS03535 (sucC)**	-3.14	succinyl-CoA ligase subunit beta	the component of succinyl-CoA synthetase involved in the citric acid cycle (TCA)
**B21_RS03530 (sucB)**	-3.28	dihydrolipoyllysine-residue succinyltransferase component of 2-oxoglutarate dehydrogenase complex	catalyzes the overall conversion of 2-oxoglutarate to succinyl-CoA and CO_2
**B21_RS03525 (sucA)**	-2.41	2-oxoglutarate dehydrogenase E1 component	the component of the 2-oxoglutarate dehydrogenase complex that catalyzes the overall conversion of 2-oxoglutarate to succinyl-CoA and CO_2
**B21_RS06035 (icdA)**	-2.92	isocitrate dehydrogenase (NADP(+))	connected with rapid shifts between TCA and glyoxalate bypass pathways
**B21_RS16115 (mdh)**	-2.21	malate dehydrogenase	catalyzes the reversible oxidation of malate to oxaloacetate
**B21_RS08295 (fumC)**	-3.48	class II fumarate hydratase	involved in the TCA cycle. FumC seems to be a backup enzyme for FumA under conditions of iron limitation and oxidative stress
**B21_RS19540 (fadA)**	-3.80	3-ketoacyl-CoA thiolase	catalyzes the final step of fatty acid oxidation
**B21_RS19545 (fadB)**	-4.74	multifunctional fatty acid oxidation complex subunit alpha	involved in the aerobic and anaerobic degradation of long-chain fatty acids via beta-oxidation cycle
**B21_RS11745 (fadI)**	-3.41	3-ketoacyl-CoA thiolase	catalyzes the final step of fatty acid oxidation
**B21_RS01140 (fadE)**	-4.76	acyl-CoA dehydrogenase	involved in the pathway fatty acid beta-oxidation which is part of lipid metabolism

**B21_RS11740 (fadJ)**	-3.69	multifunctional fatty acid oxidation complex subunit alpha	catalyzes the formation of a hydroxyacyl-CoA; strongly involved in the anaerobic degradation of long and medium-chain fatty acids
**B21_RS15365 (fadH)**	-2.89	NADPH-dependent 2.4-dienoyl-CoA reductase	involved in the beta-oxidation of unsaturated fatty acids with double bonds at even carbon positions
**B21_RS09320 (fadD)**	-3.25	long-chain-fatty-acid-CoA ligase	catalyzes the esterification. concomitant with transport. of exogenous long-chain fatty acids into metabolically active CoA thioesters for subsequent degradation or incorporation into phospholipids
**B21_RS00615 (acnB)**	-1.57	aconitate hydratase B	involved in the catabolism of short chain fatty acids (SCFA)
**B21_RS10515 gatY**	-4.03	tagatose-1. 6-bisphosphate aldolase	catalytic subunit of the tagatose-1.6-bisphosphate aldolase GatYZ. which catalyzes reversible aldol condensation
**B21_RS10510 (gatY_2)**	-2.75	tagatose-bisphosphate aldolase	component of the tagatose-1.6-bisphosphate aldolase GatYZ that is required for full activity and stability of the Y subunit
**B21_RS03685 (gpmA)**	-3.28	2.3-bisphosphoglycerate-dependent phosphoglycerate mutase	catalyzes the interconversion of 2-phosphoglycerate and 3-phosphoglycerate
**B21_RS02785 (fepA)**	-2.88	outer membrane receptor FepA	involved in the initial step of iron uptake by binding ferrienterobactin. that allows *E. coli* to extract iron from the environment
**B21_RS08660 (sufD)**	-2.82	Fe-S cluster assembly protein SufD	the component of SufBCD complex that acts synergistically with SufE to stimulate the cysteine desulfurase activity of SufS; the complex contributes to the assembly or repair of oxygen-labile iron-sulfur clusters under oxidative stress
**B21_RS11935 (mntH)**	-2.77	divalent metal cation transporter	H+-stimulated. divalent metal cation uptake system; involved in manganese and iron uptake
**B21_RS15775 (nlpI)**	-2.82	lipoprotein NlpI	probably involved in cell division and play a role in bacterial septation or regulation of cell wall degradation during cell division
**B21_RS13530 (nlpD)**	-2.78	lipoprotein NlpD	activates the cell wall hydrolase AmiC; required for septal murein cleavage and daughter cell separation during cell division
**B21_RS00070 (dnaK)**	-4.66	molecular chaperone DnaK	involved in the initiation of phage lambda DNA replication and chromosomal DNA replication
**B21_RS12940 (clpB)**	-3.20	chaperone protein ClpB	involved in the recovery of the cell from heat-induced damage. part of a stress-induced multi-chaperone system
**B21_RS20435 (aceA)**	-2.67	isocitrate lyase	involved in the metabolic adaptation in response to environmental changes
**B21_RS20430 (aceB)**	-1.99	malate synthase A	involved in step **2** of the subpathway that synthesizes (S)-malate from isocitrate
**B21_RS04955 (phoE)**	-3.77	phosphoporin PhoE	outer membrane phosphoporin PhoE that expression is induced under phosphate limitation
**B21_RS05440 (phoH)**	-2.92	phosphate starvation protein PhoH	the enzyme that possesses ATP binding activity and similarity to –terminal domain of superfamily I helicases
**B21_RS16450 (secY)**	-2.63	protein translocase subunit SecY	the central subunit of the protein translocation channel SecYEG on the extracellular side of the membrane which forms a plug
**B21_RS12705 (hmp)**	-3.10	flavohemoprotein	involved in NO detoxification in an aerobic process. termed nitric oxide dioxygenase (NOD) reaction that utilizes O_2 and NAD(P)H to convert NO to nitrate. which protects the bacterium from various noxious nitrogen compounds

**B21_RS01985 (cyoE)**	-3.60	protoheme IX farnesyltransferase	converts heme B (protoheme IX) to heme O by substitution
**B21_RS00505 (lpxC)**	-4.02	UDP-3-O-[3-hydroxymyristoyl] N-acetylglucosamine deacetylase	catalyzes the hydrolysis of UDP-3-O-myristoyl-N-acetylglucosamine to form UDP-3-O-myristoylglucosamine and acetate. the committed step in lipid A biosynthesis
**B21_RS00605 (lpdA)**	-2.75	dihydrolipoyl dehydrogenase	involved in the glycine cleavage system as well as of the alpha-ketoacid dehydrogenase complexes
**B21_RS00600 (aceF)**	-2.40	dihydrolipoyllysine-residue acetyltransferase component of pyruvate dehydrogenase complex	the component of pyruvate dehydrogenase complex catalyzes the overall conversion of pyruvate to acetyl-CoA and CO_2
**B21_RS00595 (aceE)**	-1.84	pyruvate dehydrogenase E1 component	catalyzes the overall conversion of pyruvate to acetyl-CoA and CO_2
**B21_RS06220 (dadX)**	-2.98	alanine racemase catabolic	isomerizes L-alanine to D-alanine which is then oxidized to pyruvate by DadA
**B21_RS06215 (dadA)**	-3.66	D-amino acid dehydrogenase small subunit	catalyzes the oxidative deamination of D-amino acids
**B21_RS09425 (cspC)**	-3.39	cold-shock protein CspC	involved in transcription antitermination and regulation of expression of RpoS and UspA
**B21_RS09430 (yobF)**	-3.00	DUF2527 domain-containing protein	small protein involved in stress response
**B21_RS02860 (cstA)**	-1.95	carbon starvation protein A	involved in peptide utilization during carbon starvation
**B21_RS22270 (cstA)**	-2.42	carbon starvation protein A	utilizes peptide during carbon starvation
**B21_RS12825 (rseA)**	-3.17	anti-sigma-E factor RseA	inhibits sigma-E factor that is related to stress response
**B21_RS21115 (aspA)**	-3.93	aspartate ammonia-lyase	carries out the reversible conversion of L-aspartate to fumarate and ammonia
**B21_RS16645 (tuf)**	-2.93	translation elongation factor EF-Tu 1	promotes the GTP-dependent binding of aminoacyl-tRNA to the A-site of ribosomes during protein biosynthesis
**B21_RS06855 (pspA)**	-3.67	phage shock protein A	involved in the competition for survival under nutrient- or energy-limited conditions
**B21_RS15850 (ftsH)**	-2.50	ATP-dependent zinc metalloprotease FtsH	acts as a processive. ATP-dependent zinc metallopeptidase for both cytoplasmic and membrane proteins. plays a role in the quality control of integral membrane proteins
**B21_RS16425 (rpoA)**	-2.74	DNA-directed RNA polymerase subunit alpha	catalyzes the transcription of DNA into RNA
**B21_RS18165 (kbl)**	-2.31	glycine C-acetyltransferase	catalyzes the cleavage of 2-amino-3-ketobutyrate to glycine and acetyl-CoA
**B21_RS07515**	-2.35	hypothetical protein	-
**B21_RS08995 (astB)**	-2.49	succinylarginine dihydrolase	catalyzes the hydrolysis of N2-succinylarginine into N2-succinylornithine. ammonia and CO_2
**B21_RS04930 (ldtD)**	-1.83	transpeptidase	removal of the D-alanine residue of an acyl donor peptidoglycan tetrapeptide stem
**B21_RS12900 (pka)**	-1.32	protein lysine acetyltransferase	acetylates and inactivates the acetyl-CoA synthase (Acs) and also acetylate other central metabolic enzymes in response to environmental changes
**B21_RS09010 (argD)**	-2.48	aspartate aminotransferase family protein	catalyzes amination steps in arginine and lysine biosynthesis
**B21_RS02800 (entF)**	-1.55	enterobactin synthase subunit F	activates the carboxylate group of L-serine via ATP-dependent PPi exchange reactions to the aminoacyladenylate
**B21_RS20490 (pgi)**	-1.44	glucose-6-phosphate isomerase	involved in the pathway gluconeogenesis. which is part of carbohydrate biosynthesis

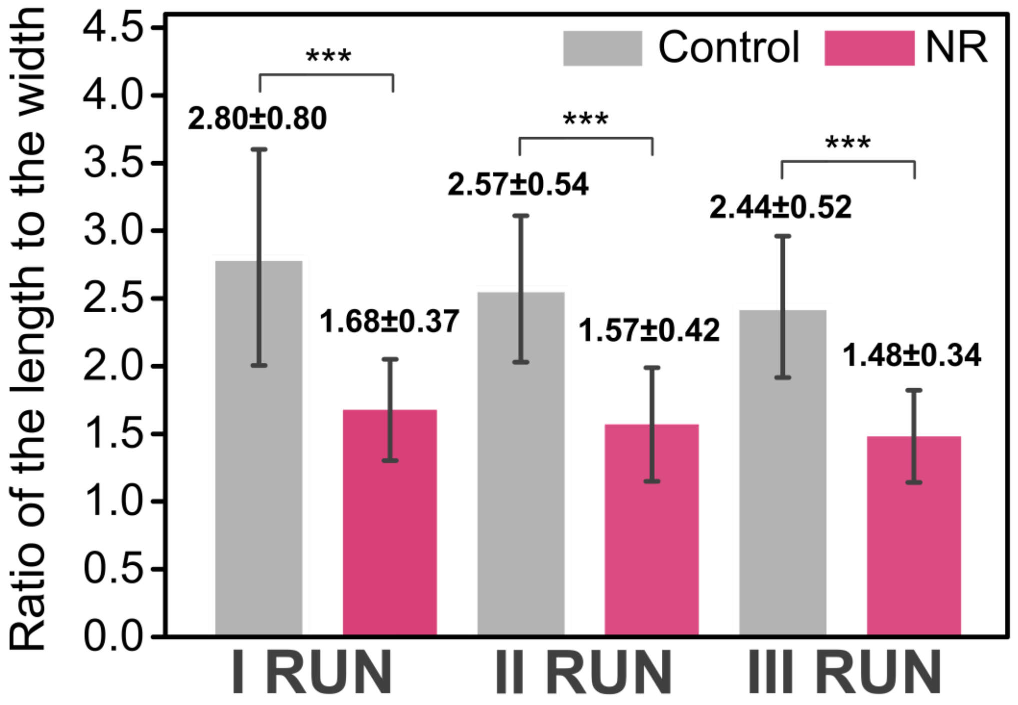

Supplementary Figure S1. The ratio of length to width of *E. coli* exposed to ZnO nanorods calculated from SEM images. Comparison of ratio on the basis of the measured length and width of control *E. coli* cells (Control) and cells after each of three exposures to ZnO nanorods (NR). Measurements were performed from three biological repeats. In each population (Control and NR) over 200 cells were measured after each exposure. Error bars show standard error of the mean (s.e.m.), where *** $P < 0.001$.

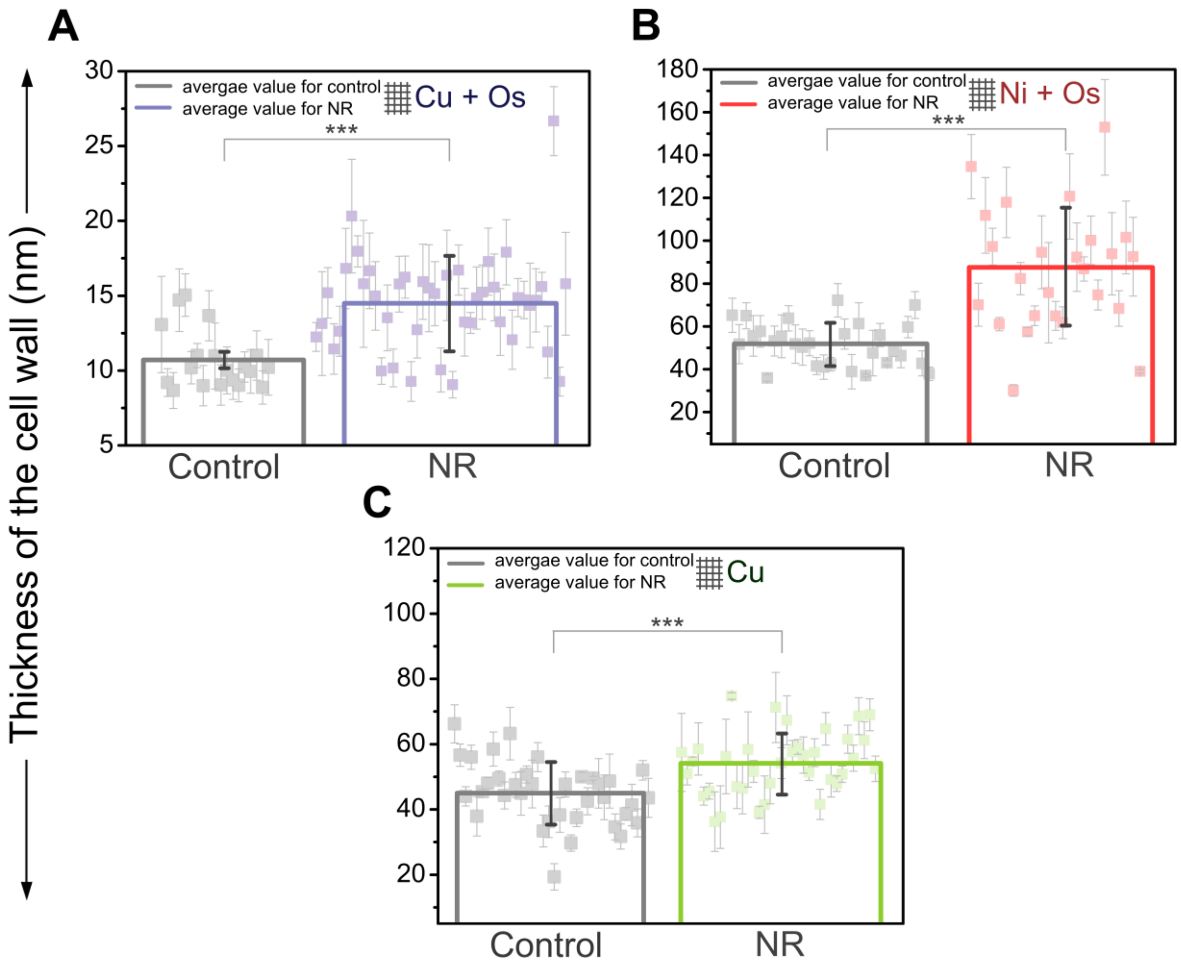

Supplementary Figure S2. The thickness of the cell wall of control *E. coli* (marked as Control) and the bacteria after one exposure to ZnO nanorods (marked as NR) measured on the basis of transmission electron microscopy (TEM) images. Single points correspond to the mean value for single bacterial cells where the cell wall was measured at three different spots. Error bars show the deviation from these three measurements for one cell. Histograms show the average thickness of the bacterial cell wall measured for the whole population of control *E. coli* bacteria and cells subjected to ZnO nanorods. Error bars show standard error of the mean (s.e.m.), where *** $P < 0.001$. (**A**) The thickness of the cell wall measured for bacteria prepared on copper meshes with osmium tetroxide saturation. The average cell wall thickness increased by 35.6 % in the case of bacteria exposed to NR (the thickest cell wall changed by 149.3%). (**B**) The thickness of the cell wall measured for bacteria on nickel meshes with osmium tetroxide saturation. The average cell wall thickness increased by 69.9% in the case of bacteria exposed to ZnO nanorods (the thickest cell wall was changed by 195.8%). (**C**) The thickness of the cell wall measured for bacteria on copper meshes. The average cell wall thickness increased by 20.8% in the case of *E. coli* subjected to mechanical stress (the thickest cell wall changed by 66.3%).

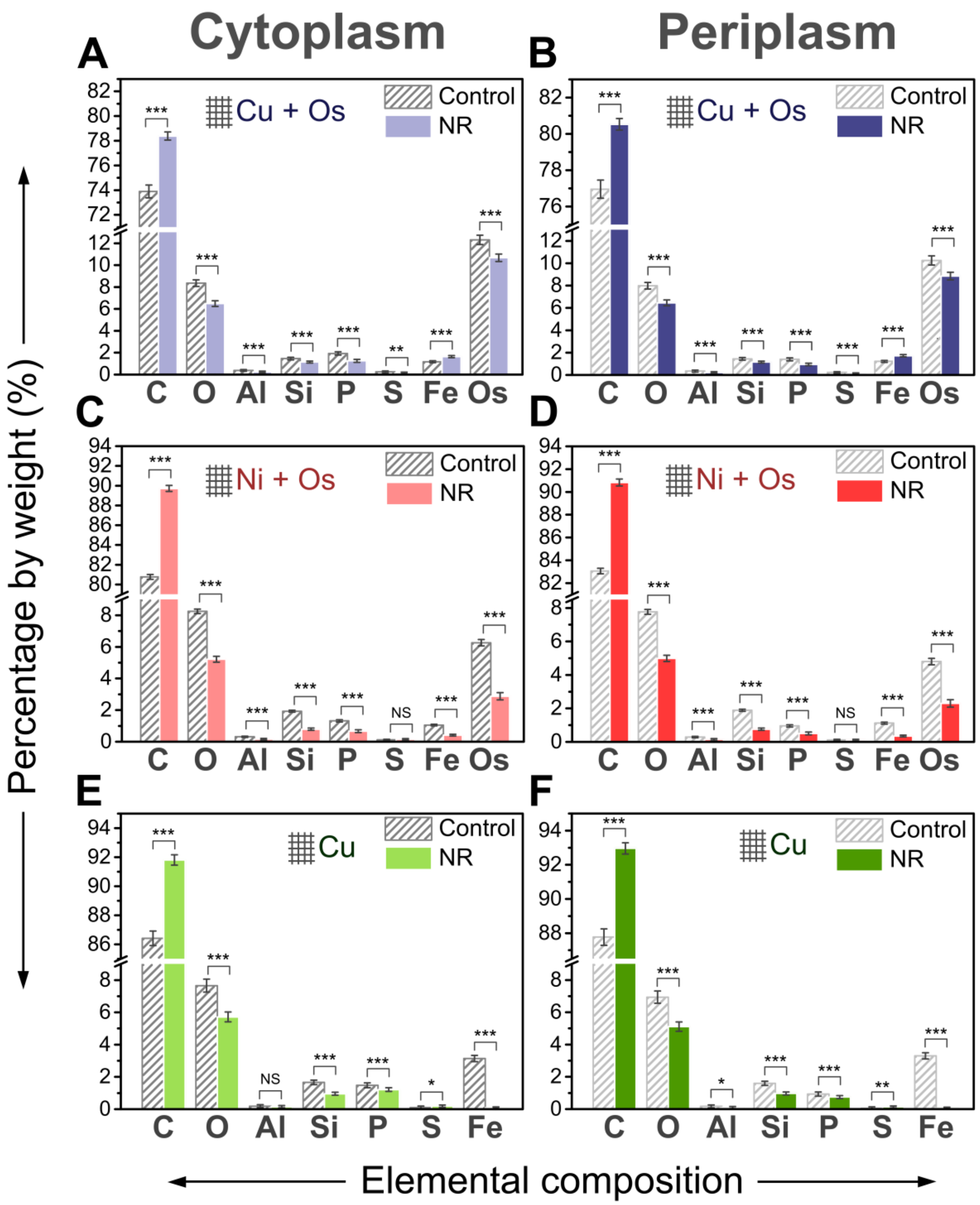

Supplementary Figure S3. Change of elemental composition of cytoplasm and periplasm of *E. coli* exposed to ZnO nanorods analyzed by energy dispersive X-ray spectroscopy (EDS). Comparison of elemental composition of control *E. coli* cells (marked as Control) and cells after exposure to ZnO nanorods (marked as NR). Error bars show standard error of the mean (s.e.m.), where * *P < 0.05; *** *P < 0.01; **** *P < 0.001*; NS, not significant. (**A**) Elemental composition of the cytoplasm of *E. coli* bacteria prepared on copper meshes with osmium tetroxide postfixation. (**B**) Elemental composition of periplasm of *E. coli* prepared on copper meshes with osmium tetroxide postfixation. (**C**) Elemental composition of the cytoplasm of *E. coli* prepared on nickel meshes with osmium

tetroxide postfixation. (**D**) Elemental composition of periplasm of *E. coli* bacteria prepared on nickel meshes with osmium tetroxide postfixation. (**E**) Elemental composition of the cytoplasm of *E. coli* prepared on copper meshes. (**f**) Elemental composition of periplasm of *E. coli* prepared on copper meshes.

2. Hertzian theory of collisions: interaction between *Escherichia coli* bacteria cells and ZnO nanorods

The interaction between bacteria and ZnO nanorods suspended in a medium can be described using contact mechanics formalism. **Supplementary Fig. S4** shows the characteristic mode of action of a ZnO nanorod (NR) on a bacterium. During stirring (200 rpm) the characteristic relative velocities of the bacteria and the nanorods are of the order of v~0.2-1.8 m s^{-1} ($v = \omega \cdot r$, v is the linear velocity of a medium in a flask in a shaker, ω is the angular viscosity and r is the distance of a moving fluid from the axis of rotation, r~1 cm to 10 cm). The size of the bacterium is d_b ~1 μm and its mass is m_b~10^{-12} g. The viscosity of water is η~1 mPas, therefore the Stokes number $Stk = m_b v/(\eta {d_b}^2)$ for the bacterium is 10^{-1}. The Stokes number compares the inertia (given by the momentum of the bacterium) to dissipation forces proportional to viscosity. For Stk<<1 the bacterium follows the streamlines of water during stirring.

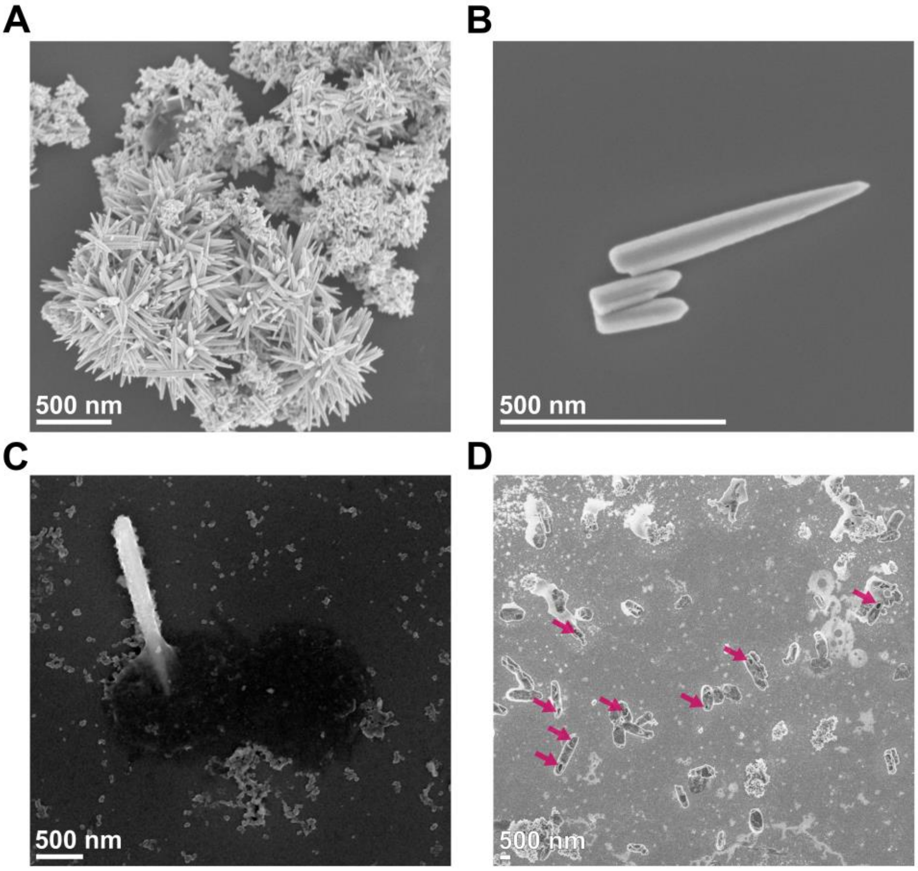

Supplementary Figure S4. Interaction between *E. coli* bacteria and ZnO nanorods. (**A**) Sharp, cone-like tips of ZnO NR. (**B**) Size of a single ZnO nanorod. (**C**) *E. coli* cell pierced by ZnO nanorods (image adapted by permission of The Royal Society of Chemistry[1]). (**D**) SEM image of a native sample of *E. coli* bacteria after exposure to ZnO nanorods. The arrows indicate holes in cells which were pierced by nanorods.

For cylindrical nanorods of length 400 nm, radius 40 nm and density ρ~5.6 g cm^{-3} (ZnO) the mass is m_{nr}~10^{-14}g, giving Stk~10^{-4} for the nanorods. Thus, they also follow the streamlines of water. However, the Reynolds number $R_e = \rho v L/\eta$~10^4 (for density of water at 37 °C ρ~10^3 kg m^{-3} and dynamic viscosity η~7·10^{-4} Pa·s, moving in a flask of L~0.1 m with velocity v~1 m s^{-1}), thus the flow of the medium is turbulent and promotes high velocity collisions of the bacteria with the nanorods. Upon direct contact, when the bacteria and nanorods collide the pressure exerted at the surface of the bacterial cell is large enough to pierce it as shown in **Supplementary Fig. S4C**. We estimate this pressure using the Hertzian theory of collisions, since eventually, both objects come into mechanical contact. The characteristic time of collision is given by the equation[2]:

$$t = 2.54\rho^{2/5}\left(\frac{1-\mu_{nr}^2}{E_{nr}} + \frac{1-\mu_b^2}{E_b}\right)^{2/5} dV^{-1/5} \qquad (1)$$

Where E is the Young modulus of the nanorods (nr) and the bacteria (b) and μ is the Poisson ratio, d is the size of contact (size of the tip of the nanorod) and V is the velocity upon impact. The Young modulus of the bacteria is E_b~ 25 MPa[3] and is four orders of magnitude smaller than the Young modulus of NR E_{nr}~100 GPa[4]. The Poisson ratio for the bacteria is ~0.4[5]. The size of the tip of the nanorod obtained from SEM measurements is 10 nm, but we suspect that the tip is rough and locally we may have 1 nm roughness. For d=10 nm, we find the collision time t=1 ns. The force exerted by the 10 nm tip on the bacterial surface is $F = m_{nr} \cdot V/t$ =1 nN and 10 nN for the 1 nm tip. The pressure exerted at the surface of the bacteria is also given by the Hertz theory[6]. For a cylindrical tip of radius d/2 acting with a force F on the bacteria, from the Hertz theory, we have $p = 2F/\pi d^2$~0.1 MPa (for collisions with sides of the nanorod) to

10^4 MPa (for direct collisions with the 1 nm tip). This lowest pressure is comparable to the turgor pressure inside the bacteria (~0.3 MPa[7]) and the larger one is much greater than the Young modulus of bacteria. This pressure may be much larger if we consider a cone geometry instead of a cylinder[6]. In this case, the pressure theoretically diverges at the tip of the cone. Thus, the physical effect of the mechanical stress induced by the nanorods should strongly affect the bacteria and they could be hit many times without losing their viability.

3. Viability curves of *Escherichia coli* bacteria exposed to ZnO nanostructures

Escherichia coli cells were subjected to stress induced by two types of ZnO nanostructures of the same concentration and a similar active surface area: sharp nanorods and shapeless, nearly spherical nanostructures (NP). The shape of the ZnO nanoparticles was utilized as a marker indicating changes within the cell envelope of the bacteria. The difference between the viability curves of Gram-negative bacteria exposed to NR and NP during the first exposure is presented in **Supplementary Fig. S5A**. Comparison of viability curves after the second exposure to NR and NP revealed that *E. coli* became invulnerable to both types of nanostructures, i.e. no difference between viability after exposure to NR or NP was observed (**Supplementary Fig. S5B**). The lack of susceptibility to the shape of the nanostructures was a characteristic feature observed for Gram-positive strains (*Staphylococcus epidermidis*, *Corynebacterium glutamicum*) that possess more peptidoglycan in the cell wall than Gram-negative bacteria. This specific feature acquired by *E. coli* exposed to NR was the first sign of changes occurring within the cell wall.

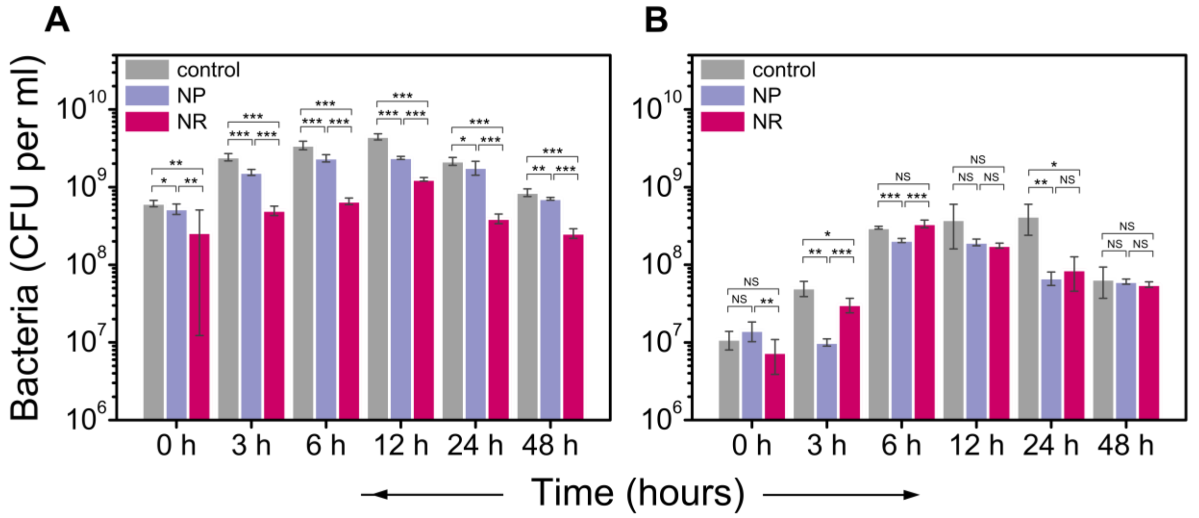

Supplementary Figure S5. Viability curves of *E. coli* after exposure to sharp nanorods (NR) and rounded nanostructures (NP). (**A**) The first exposure to the nanostructures (image adapted by permission of The Royal Society of Chemistry [1]). (**B**) The second exposure of *E. coli* that had survived the first exposure to NR. Error bars show standard error of the mean (s.e.m.), where * $P < 0.05$; ** $P < 0.01$; *** $P < 0.001$; NS, not significant.

Procedure: An overnight culture was prepared by the inoculation of a single colony of *E. coli* BL21(DE3) (bacteria obtained from the Institute of Biochemistry and Biophysics, Polish Academy of Science in Warsaw, Poland) into LB medium (Roth) for seven hours (37 °C, 200 rpm). Chloramphenicol and kanamycin (Sigma-Aldrich) were added to the medium to a final concentration of 50 μg ml^{-1} and 25 μg ml^{-1}, respectively. 100 μl of the overnight culture was taken, inoculated in the fresh LB medium and cultured to obtain a suspension of OD_{600} = 0.5 (optical density). The bacterial suspension was divided into three sterile flasks. Just before the experiment, ZnO NR were suspended in 1 ml of medium and added to one flask to obtain a final concentration of 1 mg ml^{-1}. ZnO NP were suspended in the same way. 1 ml of pure LB was added to the control flask to maintain the same starting amount of bacteria in all three flasks. Bacteria were cultured in a shaker (37 °C, 200 rpm; IKA KS 4000 i, Germany) for 48 hours. At each time point, 100 μl was taken to prepare a series of dilutions and 50 μl of each dilution was plated on LB agar plates. At least six technical replicates were performed for each dilution. The plates were incubated for 24 hours at 37 °C in an incubator (Binder, Germany).

The colony count method was applied to determine the viability curves. After 24 hours, 100 µl of the bacteria that had survived the first exposure to sharp ZnO nanorods were inoculated in a fresh portion of LB medium for overnight culturing. The bacterial suspension was refreshed to reach OD_{600} = 0.1. The second exposure to NR and NP was performed analogously to the protocol described above. The experiment was conducted in three biological repeats.

4. Dependence of the viability curves on the initial inoculum of *E. coli*

E. coli were exposed to ZnO nanorods at a concentration of 1 mg ml^{-1}. The difference between the viability curves was examined during six hours of exposure in experiments with a different initial number of bacterial cells (**Supplementary Fig. S6**). With a decreasing initial number of bacteria, the difference between the control bacteria and the cells exposed to nanorods was more pronounced at the beginning of the experiment. At the same concentration of nanorods and a decreasing number of cells, there were more nanorods per bacterium. This resulted in a higher pressure being induced on *E. coli* cells at the start of the experiment.

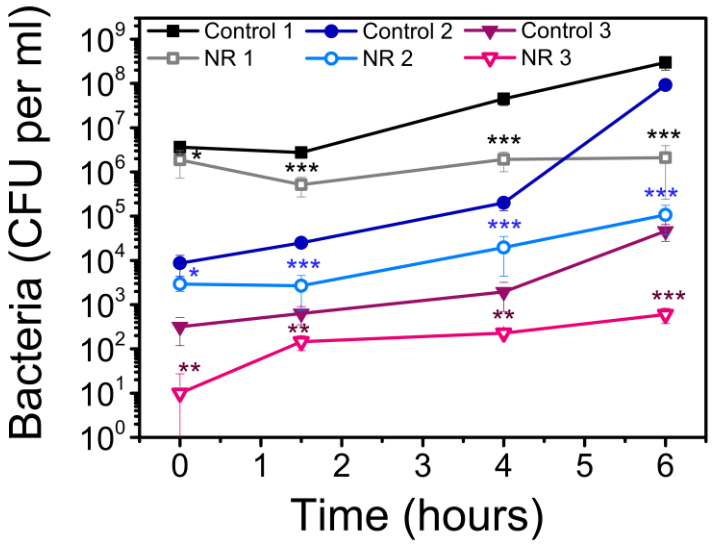

Supplementary Figure S6. Viability curves of *E. coli* after exposure to sharp nanorods (NR). Error bars show the standard error of the mean (s.e.m.), where * *P < 0.05; *** *P < 0.01;* *** *P < 0.001*. In this case, control bacteria and cells after exposure to NR are compared in pairs (e.g. Control 1 vs NR 1).

Procedure: Experiments were performed for *E. coli* BL21 according to the protocol described in the previous section. The main difference was that at the beginning of the experiment a bacterial suspension of $OD_{600} = 0.1$ was used to prepare three initial suspensions of bacteria of concentrations ~$5 \cdot 10^6$, 10^4 and $5 \cdot 10^2$ bacteria per ml. The exposure was conducted for six hours. The colony count method was used to determine the number of bacterial cells. At least six technical replicates were performed for each time point. The experiment was performed in two biological repeats.

5. Supplementary comparison of UV-Vis spectra after Gram staining of *E. coli* exposed to ZnO nanorods and spherical ZnO nanoparticles

Gram staining is a classical, microbiological procedure used to distinguish two groups of bacteria: Gram-negative and Gram-positive strains, based on the thickness of peptidoglycan within the cell wall.

It appeared that survivor *E. coli* bacteria (after the first exposure) which were subjected to a second exposure became invulnerable to mechanical collisions with the nanorods. **Supplementary Fig. S7** depicts a chemical interpretation of Gram staining (comparison of UV-Vis spectra) that is an extended version of **Fig. 1B** from the main text. Here, the UV-Vis spectrum for *E. coli* bacteria after exposure to spherical nanoparticles is shown additionally. The procedure for Gram staining is described in the main text in the Section: UV-Vis spectra of Gram-stained bacteria. The exposure of bacteria to spherical nanoparticles was performed analogically to the exposure to ZnO NR.

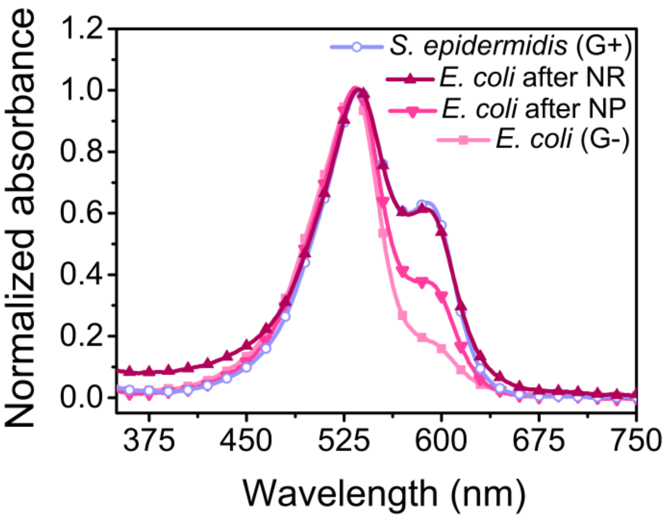

Supplementary Figure S7. Comparison of UV-Vis spectra of samples after Gram staining. Control *E. coli* and *S. epidermidis* are marked *E. coli* (G-) and *S. epidermidis* (G+), respectively. *E. coli* after exposure to ZnO nanorods is marked as *E. coli* after NR and *E. coli* after exposure to the more spherical ZnO nanoparticles is marked as *E. coli* after NP. The peak in the range about 530 nm corresponds to the presence of safranine. The maximum absorption for crystal violet which is within the layer of peptidoglycan is around 590.

6. Change of shape of *Enterobacter aerogenes* (Gram-negative) after exposure to ZnO nanorods

Enterobacter aerogenes (Gram-negative) and *Corynebacterium glutamicum* (Gram-positive) were exposed to ZnO nanorods to examine whether similar changes of shape were observed as in the case of *E. coli* bacteria. *E. aerogenes* and *C. glutamicum* were exposed to NR once (24 hours) and then the survivor bacteria were exposed a second time to ZnO nanorods. Next, the shape of the bacteria was analyzed using a scanning electron microscope (SEM). *E. aerogenes*, a representative of a Gram-negative strain with a thin cell wall, became spherical after exposure to ZnO nanorods (**Supplementary Fig. S8B**). *C. glutamicum* (a bacterium with a higher amount of peptidoglycan in the cell envelope) did not change shape, due to a more mechanically stable cell wall (**Supplementary Fig. S8D**).

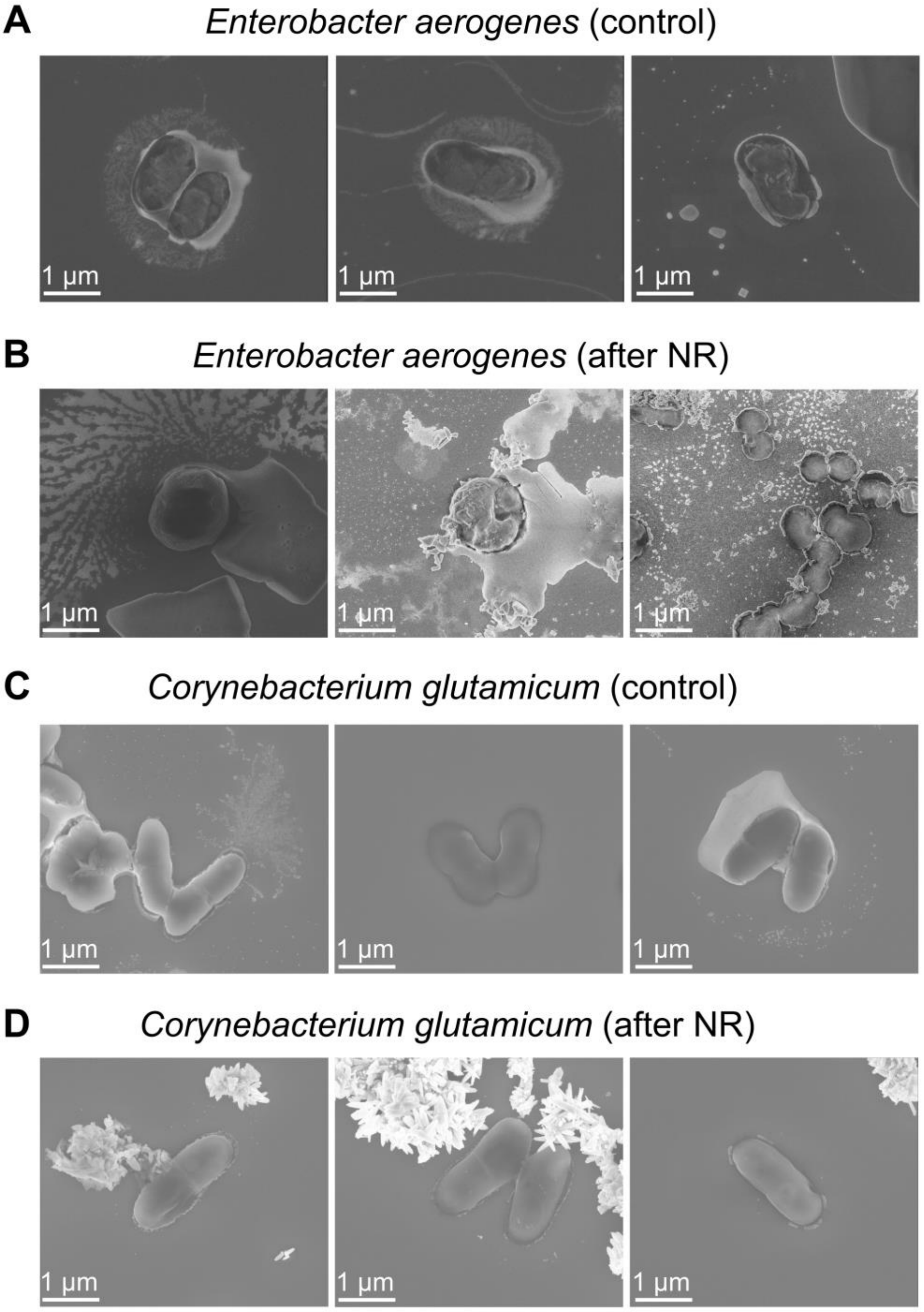

Supplementary Figure S8. Change of shape of *E. aerogenes* and *C. glutamicum* exposed to ZnO nanorods. Images of non-treated bacteria (control) and the cell exposed to ZnO nanorods (after NR) obtained by scanning electron microscopy (SEM). (**A**) Images of non-treated *E. aerogenes*. (**B**) Images of *E. aerogenes* after exposure to ZnO nanorods. (**C**) Images of non-treated *C. glutamicum*. (**D**) Images of *C. glutamicum* after exposure to ZnO nanorods.

Procedure: An overnight culture was prepared by inoculation of a single colony of *Enterobacter aerogenes* PCM 1832 or *Corynebacterium glutamicum* PCM 1954 (both strains were purchased from the Polish Collection of Microorganisms, Wrocław, Poland) into LB medium for seven hours (37 °C, 200 rpm). 100 µl of the overnight culture was taken, inoculated in the fresh LB medium and cultured to obtain a suspension of $OD_{600} = 0.1$. The bacterial suspension was divided into two sterile flasks. Just before the experiment, ZnO NR were suspended in 1 ml of medium and added to one flask to obtain a final concentration of 1 mg/ml. 1 ml of pure LB medium was added to the other flask. Bacteria were cultured in a shaker (IKA KS 4000 i, Germany) for 24 hours (37 °C, 200 rpm). After 24 hours, 100 µl of control bacteria were inoculated in a fresh portion of LB medium and similarly 100 µl of the cells that had survived the first exposure to sharp ZnO nanorods (NR) were inoculated in a fresh portion of LB medium with the nanorods. Next, the samples were prepared for further SEM analysis according to the protocol described in the main text (Material and Methods, section: Scanning electron microscopy (SEM) and cryo-scanning electron microscopy (Cryo-SEM)).

7. Supplementary transmission electron images (TEM) of *Escherichia coli* bacteria exposed to ZnO nanorods

The thickness of the bacterial cell wall after one exposure to ZnO nanorods (24 hours) was analyzed using TEM. The samples for microscopic observation were prepared in four different ways. Exemplary images of the bacteria prepared on nickel meshes and nickel meshes with osmium tetroxide saturation are shown in **Supplementary Fig. S9**. The procedure and method of determination of the cell wall thickness are described in the main text (Material and methods, section: Transmission electron microscopy (TEM); see also **Fig. 2A** and **Fig. 3B**).

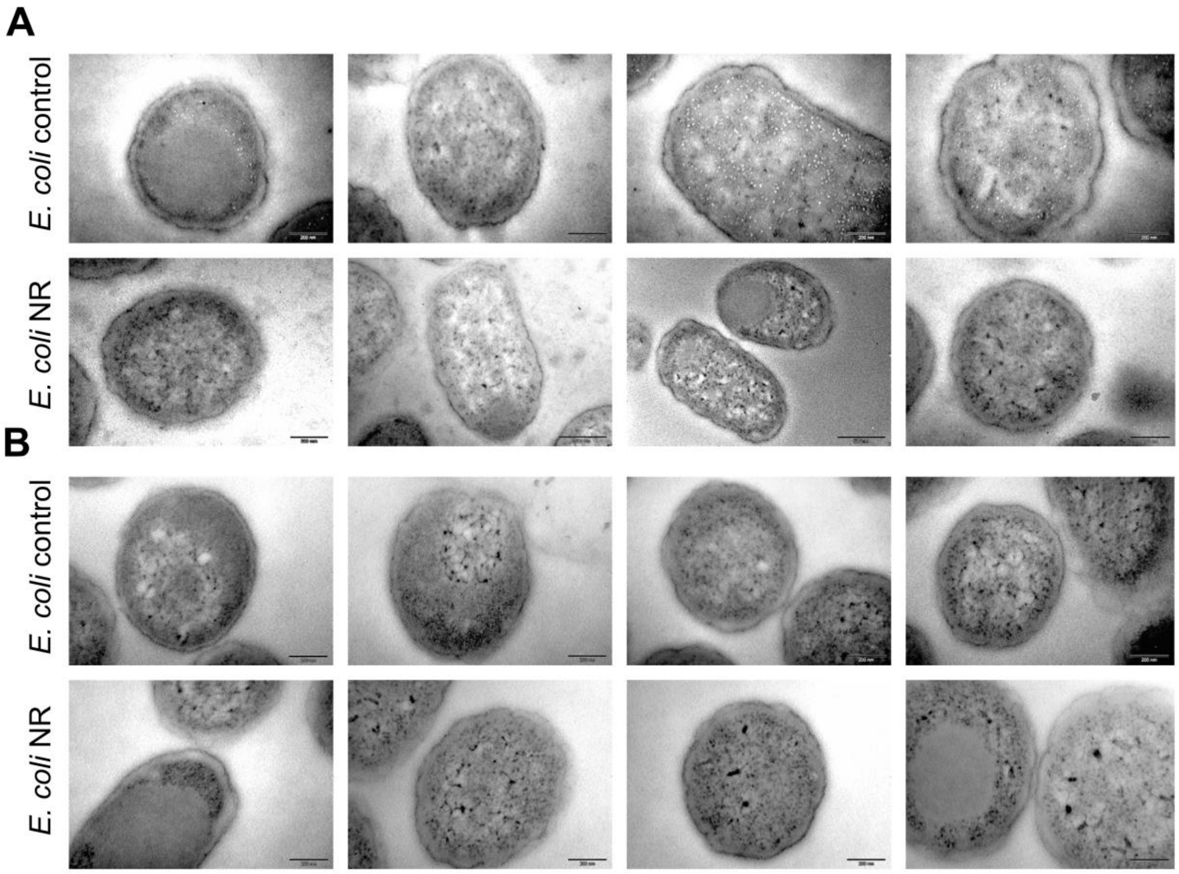

Supplementary Figure S9. TEM images of *E. coli* after one exposure to sharp nanorods (NR). The small portions of cells of each type of sample after one exposure were multiplied in the medium without nanoparticles. In the pictures, *E. coli* control cells are marked as *E. coli* control and *E. coli* cells after one exposure to ZnO nanorods are marked as *E. coli* NR. (**A**) TEM images of the bacteria prepared on nickel meshes. (**B**) TEM images of the bacteria prepared on nickel meshes with osmium tetroxide saturation.

8. Examination of transcriptome by RNA sequencing.

RNA-seq analysis revealed that the padj<0.05 criterion (modified p-value corrected by multiple testing of the differences in gene expression using the Benjamini-Hochberg procedure[8]) was fulfilled by 606 genes: 363 genes were up-regulated and 243 genes were down-regulated. **Supplementary Fig. S10-S15** show changes of expression for all up-regulated and down-regulated genes.

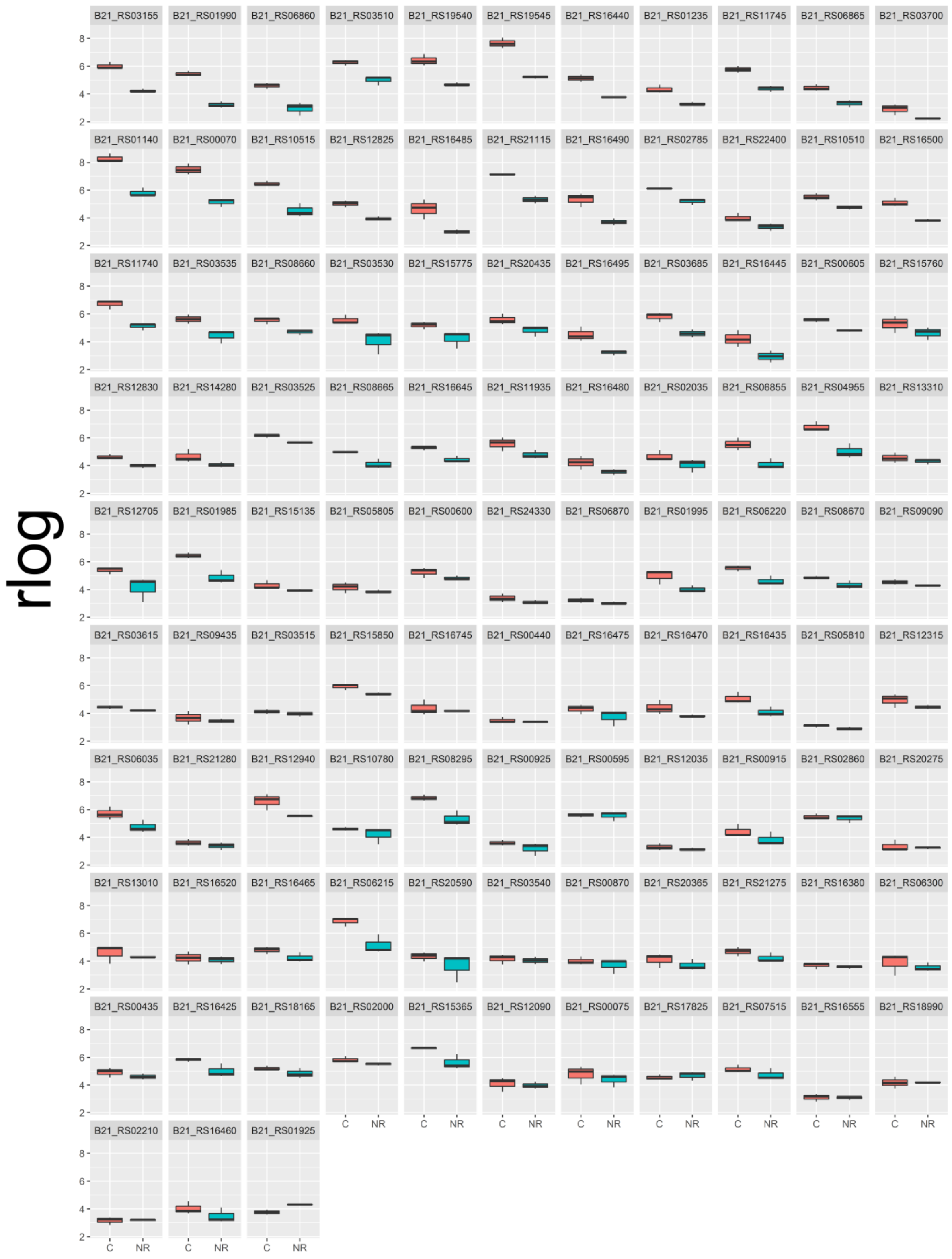

Supplementary Figure S10. Change of gene expression profile of the bacteria exposed to ZnO nanorods. Control *E. coli* bacteria (marked as C) and the cell exposed to ZnO nanorods (marked as NR). Results show a comparison of expression of genes with 4.78E-18 ≤padj≤ 0.000286991.

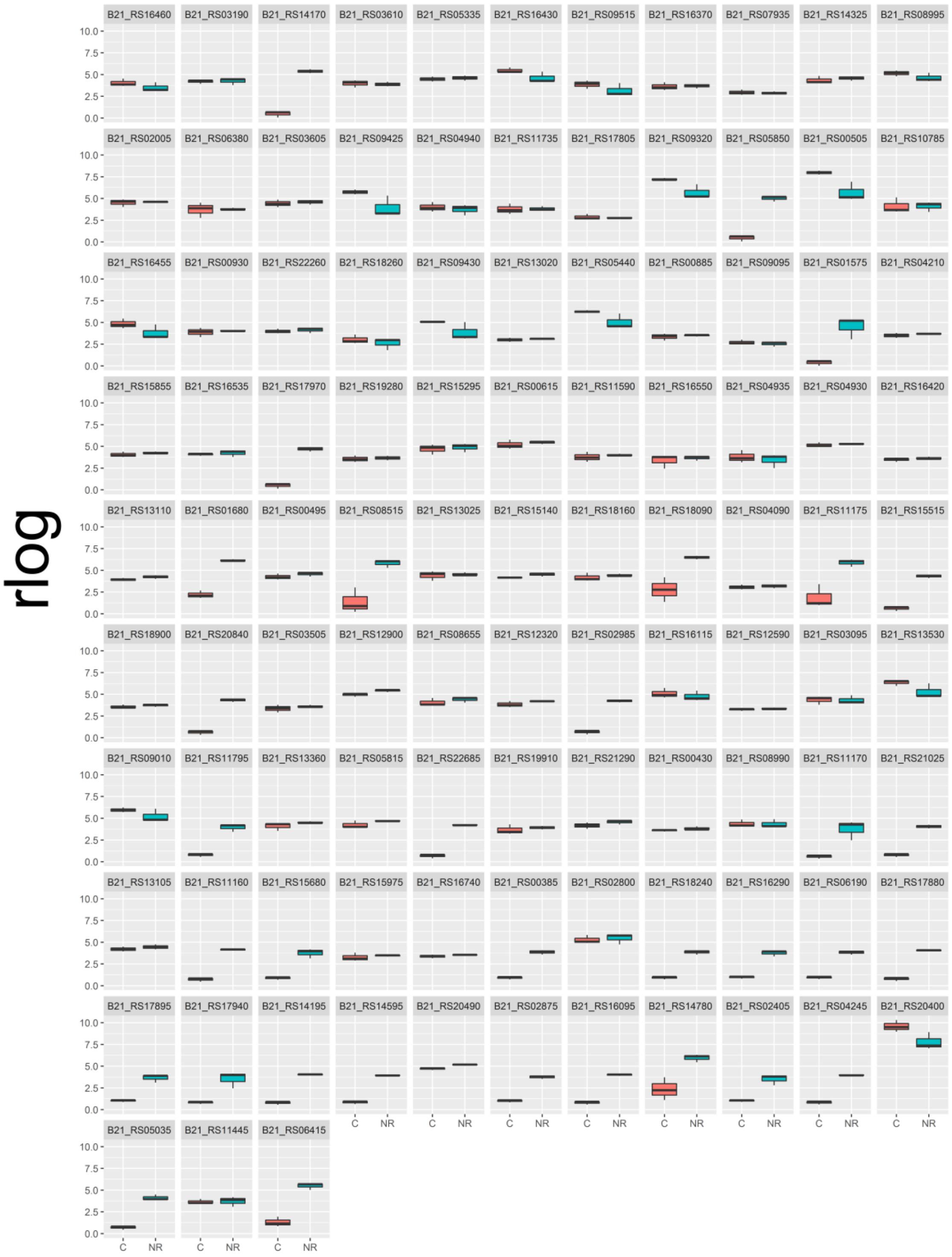

Supplementary Figure S11. Change of gene expression profile of the bacteria exposed to ZnO nanorods. Control *E. coli* bacteria (marked as C) and the cell exposed to ZnO nanorods (marked as NR). Results show a comparison of expression of genes with $0.000286991 \leq padj \leq 0.011253454$.

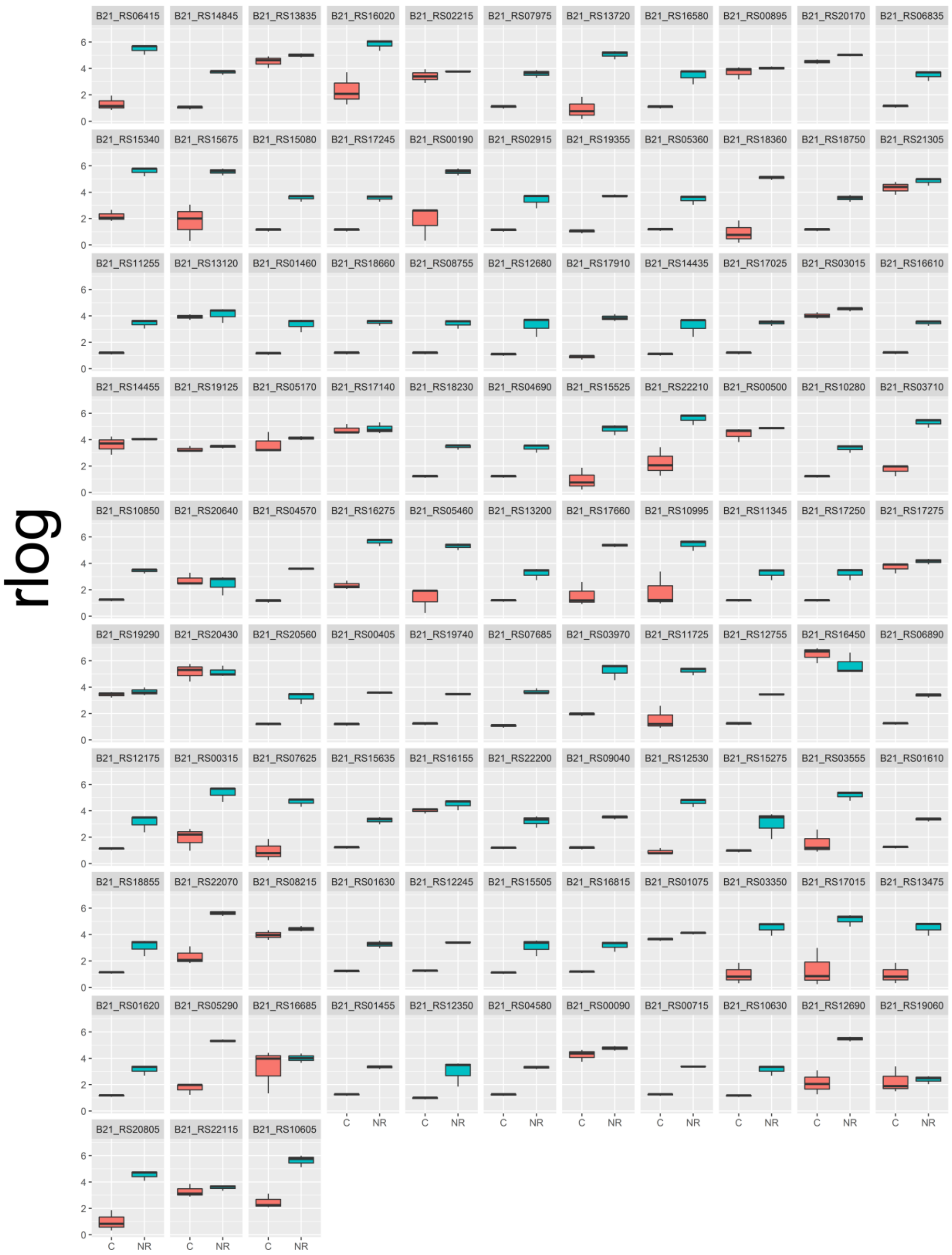

Supplementary Figure S12. Change of gene expression profile of the bacteria exposed to ZnO nanorods. Control *E. coli* bacteria (marked as C) and the cell exposed to ZnO nanorods (marked as NR). Results show comparison of expression of genes with 0.011253454≤padj≤0.023850404.

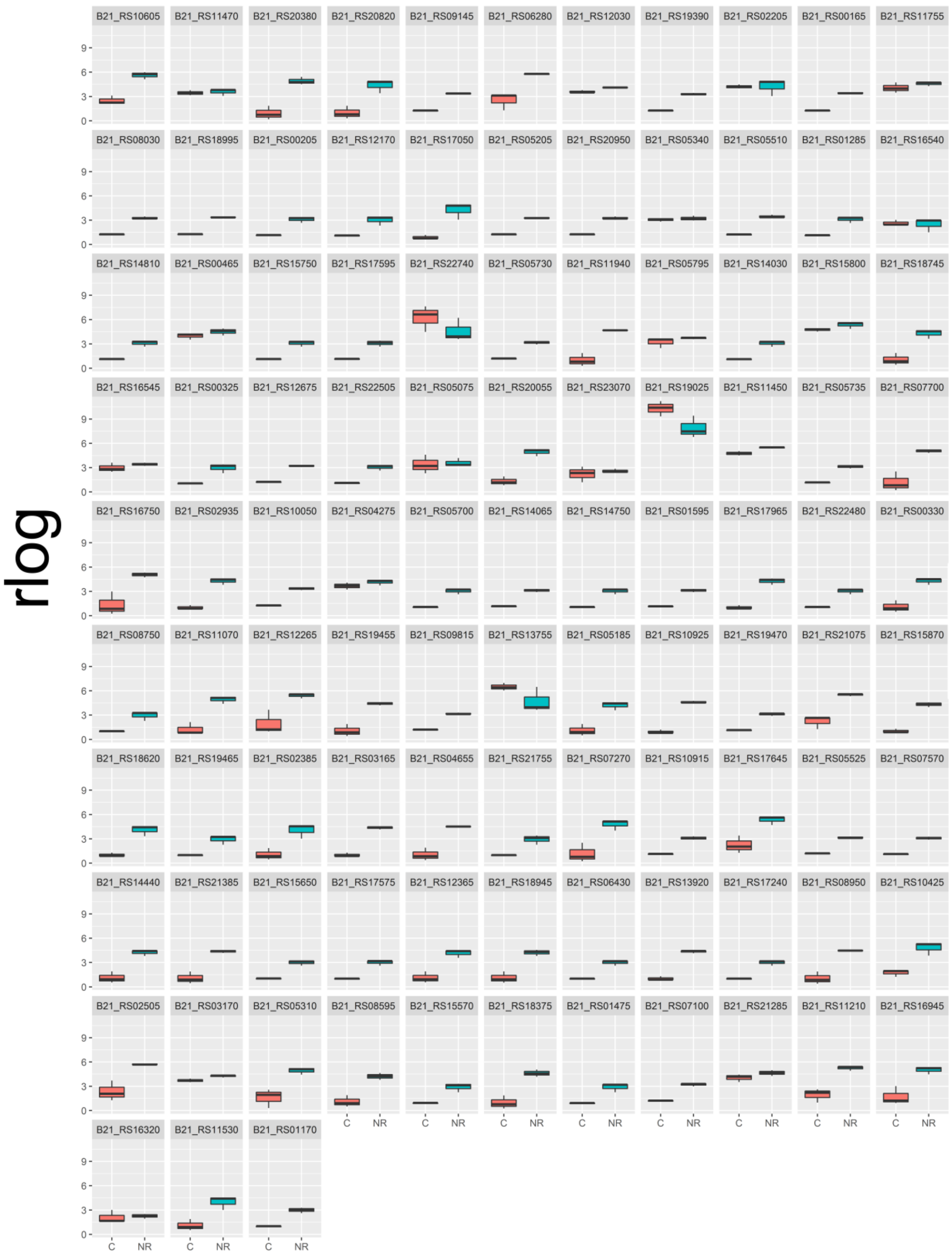

Supplementary Figure S13. Change of gene expression profile of the bacteria exposed to ZnO nanorods. Control *E. coli* bacteria (marked as C) and the cell exposed to ZnO nanorods (marked as NR). Results show a comparison of expression of genes with $0.023850404 \leq padj \leq 0.035008568$.

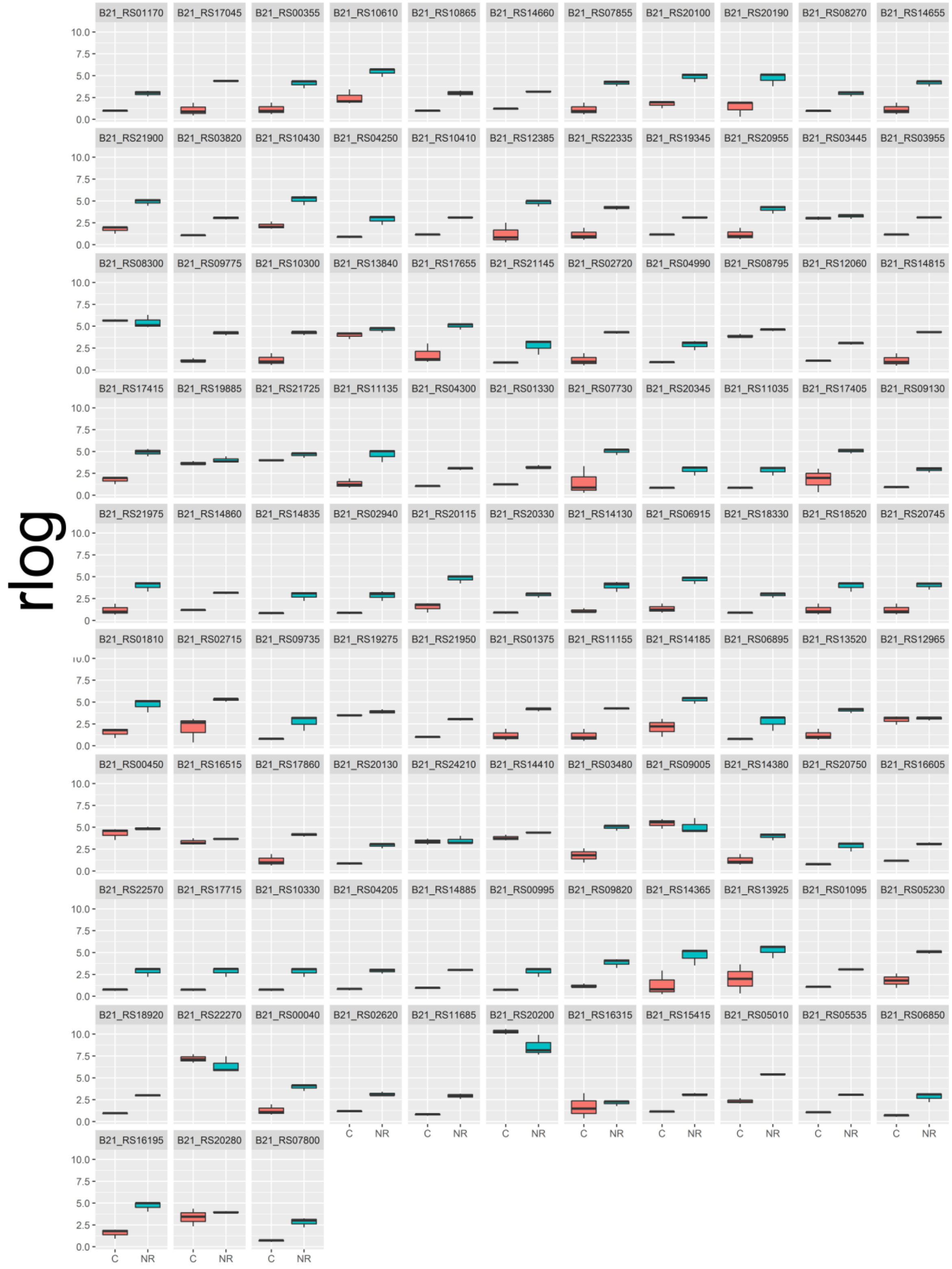

Supplementary Figure S14. Change of gene expression profile of the bacteria exposed to ZnO nanorods. Control *E. coli* bacteria (marked as C) and the cell exposed to ZnO nanorods (marked as NR). Results show a comparison of expression of genes with 0.035008568≤padj≤0.041854951.

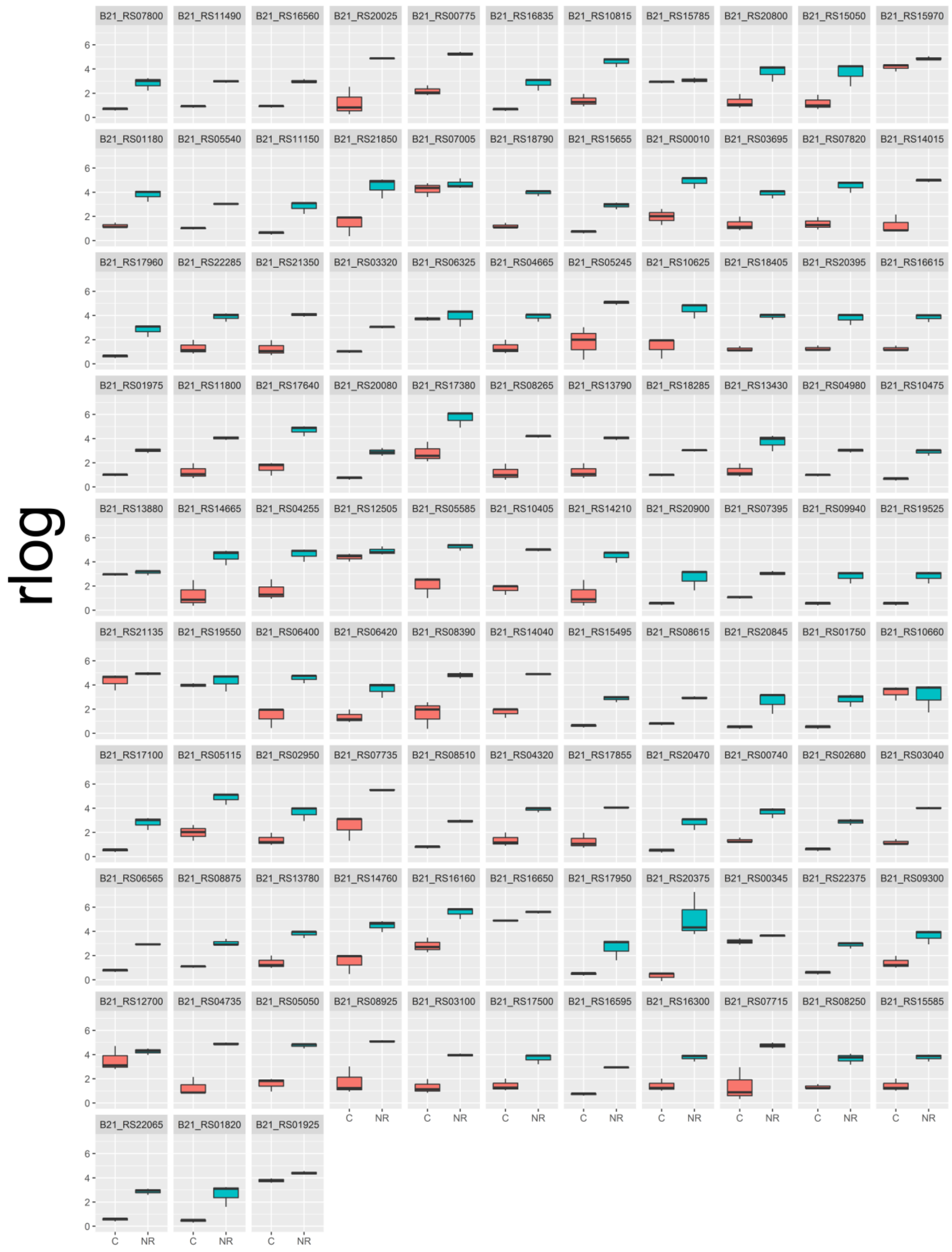

Supplementary Figure S15. Change of gene expression profile of the bacteria exposed to ZnO nanorods. Control *E. coli* bacteria (marked as C) and the cell exposed to ZnO nanorods (marked as NR). Results show a comparison of expression of genes with 0.041854951≤padj≤0.049983382.

9. Testing of phage susceptibility of *E. coli* exposed to ZnO nanorods

Bacteriophages (phages; viruses whose hosts are bacteria) were used to examine whether modification of the cell wall of *E. coli* upon exposure to ZnO nanorods affected the phage susceptibility, defined as the number of viral plaques formed (observed by the classical plaque count method). During this experiment, the morphology of the formed plaques was also analyzed. Any change in size, shape, turbidity, the appearance of the plaque halo or border of the plaques could be a sign of the handicapped/restricted mechanism of activity of the bacteriophages. We tested seven different types of bacteriophages: T7, T1, P1, λ, T4D, T4*rII*, and T4*rIII*. They represent different families of *E. coli* phages with distinct adsorption and development mechanisms. The series of T4 mutants additionally show different mutation affecting the holin functionality. In the case of the T1 phage, the formed plaques on *E. coli* bacteria after exposure to ZnO nanorods were ~40% smaller in size in comparison with plaques formed on the control bacteria. No changes in the morphology of plaques of T7, P1, λ, T4D, T4r*II*, T4r*III* phages were observed. Even if some differences between the numbers of plaques were statistically significant, they were not reflected in the case of the second examined strain (**Supplementary Fig. S16**).

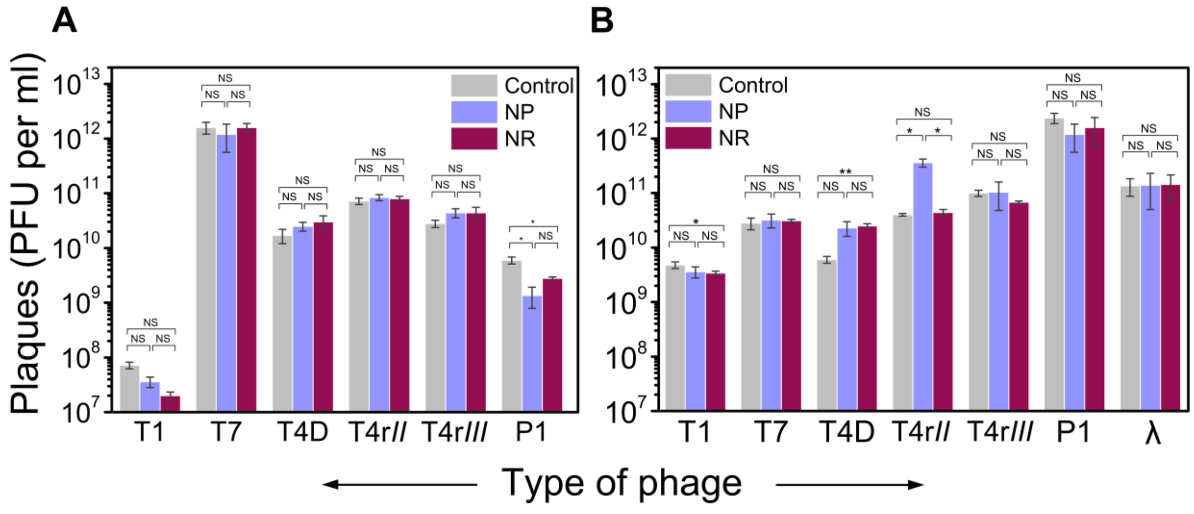

Figure S16. Formation of plaque of T7, T1, P1, λ, T4D, T4rII, T4rIII. *E. coli* without exposure to NR are

marked as control, *E. coli* cells after exposure to ZnO nanorods are marked as NR and *E. coli* cells after exposure to ZnO rounded nanoparticles are marked as NP. (**A**) The difference in the number of plaque forming units (PFU) for *E. coli* BL21(DE3). (**B**) The difference in the number of PFU for BL21. Error bars show standard error of the mean (s.e.m.), where * $P < 0.05$; ** $P < 0.01$; NS, not significant.

Procedure: Phage lysates (T7, T1, P1, λ, T4D, T4rII, T4rIII) were prepared by an infection in an early logarithmic culture of *Escherichia coli* MG1655. After bacterial lysis, the phages were precipitated by polyethylene glycol and subsequently centrifugation was performed. The precipitate was resuspended in TM buffer (10 mM Tris-HCl with 10 mM $MgSO_4$, pH = 7.4) with 1 M NaCl and purified by ultracentrifugation (Beckman Optima XL70 ultracentrifuge with Ti50 rotor) in step cesium chloride gradient. After purification, the phages were collected by aspiration using a syringe (white band in the centrifuge tube) and dialyzed against TM buffer. Then 0.2 μg ml^{-1} of DNase I was added in order to digest the DNA released from the capsids of damaged phages. The plaque count method was used to determine the number of bacteriophages in the samples and also to analyze the morphology of the plaques. The plaque forming units (PFU) were directly correlated with the number of active phages within the sample. In this experiment two bacterial strains were used: *E. coli* BL21 and BL21(DE3) in three variants: control cells, bacteria exposed to ZnO nanorods (NR) and bacteria exposed to rounded nanoparticles (NP). First, three days before the experiment an overnight culture was prepared by inoculation of a single colony of *E. coli* into LB medium for seven hours (37 °C, 200 rpm). 100 μl of the overnight culture was taken, inoculated in a fresh LB medium and cultured to obtain a suspension of $OD_{600} = 0.1$. The bacterial suspension was divided into three sterile flasks. Just before the experiment, ZnO NR and NP were suspended in 1 ml of medium and added to the sterile flasks to obtain a final concentration of 1 mg ml^{-1}. 1 ml of pure LB medium was added to the control flask. Bacteria were cultured in a shaker for 24 hours (37 °C, 200 rpm). After 24 hours, 100 μl of control bacteria were inoculated in a fresh portion of LB

medium, 100 µl of the bacteria that had survived the first exposure to sharp ZnO nanorods (NR) were inoculated in a fresh portion of LB medium with NR and 100 µl of the cells that had survived the first exposure to NP were transferred into a flask with fresh LB medium and NP. After 24 hours of the second exposure, the bacteria were subjected to a third exposure that was conducted analogously to the previous one.

On the day of the experiment, the bottom agar was prepared by pouring 25 ml of LB agar medium into sterile plastic Petri dishes. Next, 4 ml of top LB-agar was mixed with 200 µl of the bacteria (control and after three exposures to NR and NP) and 25 µl of prepared phage solution (to obtain around 100 plaques in the case of the control samples). The top LB agar consisted of LB medium and 0.75% agar. After vigorous mixing, the medium with the bacteria and the phages was immediately poured onto the dried bottom agar. The plates were incubated at 37 °C for 24 h. The plaques were counted and the morphology of the formed viral plaques was analyzed.

10. Growth kinetics of T4 bacteriophage in *E. coli* exposed to ZnO nanorods.

Bacteriophages perform a few consecutive processes within the replication cycle. They recognize specific cells, inject genetic material, and use the host cell to produce proteins and more copies of nucleic acids. Finally, new, assembled virions are released by disruption of the bacterial cell[9]. When new phages are released to an environment where not yet infected bacteria are present, the whole process is repeated, resulting in a high yield of bacteriophage replication. The cascade is repeated until all the bacteria are infected. We checked the difference in growth kinetics of T4 bacteriophage in *E. coli* BL21 after three exposures to ZnO nanorods (NR) and ZnO nanoparticles (NP) compared with non-treated cells. **Supplementary Fig. S17** compares the kinetics of growth of T4 phages in the population of control bacteria and after the cells after exposure to ZnO nanostructures. Handicap or prolongation of any step of a bacterial infection

should result in a decrease in growth rate. Such a difference was not observed. This proved no difference in growth kinetics between control bacteria and the cells exposed to NR or NP so unsynchronized replication cycle of T4 phage is not disrupted.

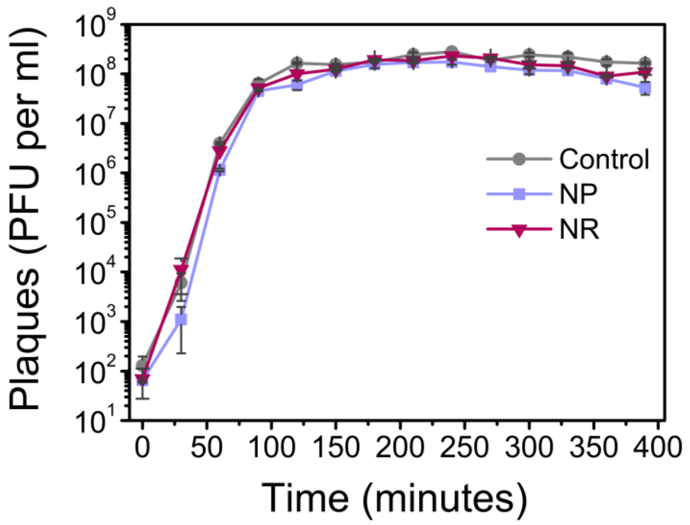

Supplementary Figure S17. Growth kinetics of T4 bacteriophage. The difference in the number of plaque forming units (PFU) for the population of control *E. coli* are (marked as control), *E. coli* cells after exposure to ZnO nanorods are marked as NR and *E. coli* cells after exposure to ZnO rounded nanoparticles are marked as NP. Error bars show standard error of the mean (s.e.m.). No statistical difference was observed.

Procedure: The experiment consisted of four main steps: 1) preparation of a bacterial suspension; 2) three exposures of bacteria to ZnO nanorods and nanoparticles; 3) refreshment of the bacterial culture; 4) evaluation of the growth kinetics of the T4 bacteriophage. First, an overnight culture of *E. coli* BL21 was prepared by inoculation of a single colony into LB (37 °C, 180 rpm). 100 µl of the overnight culture was inoculated in the fresh LB medium and cultured to obtain a suspension of OD_{600} = 0.1. The bacterial suspension was divided into three sterile flasks. Just before the experiment, ZnO nanorods and rounded nanoparticles were suspended in 1 ml of medium and added to the appropriate flasks to obtain a final concentration of 1 mg ml^{-1}. 1 ml of sterile LB medium was added to the control flask. Bacteria were cultured

in a shaker (37 °C, 200 rpm; IKA KS 4000 i, Germany or Grant OLS200). After 24 hours, 100 μl of the control bacteria were inoculated in a fresh portion of LB medium, 100 μl of the bacteria that had survived the first exposure to sharp ZnO nanorods (NR) were inoculated in a fresh portion of LB medium with NR and 100 μl of the cells that had survived the first exposure to NP was transferred into a flask with fresh LB medium and NP. After 24 hours of the second exposure, bacteria were subjected to a third exposure which was conducted in the same manner. On the day of the experiment, the bottom agar was prepared for phage titration by pouring 25 ml of LB agar medium into sterile plastic Petri dishes. Just before evaluation of the growth kinetics, the cultures were refreshed by diluting the suspension of bacteria in LB medium (volume ratio 1:100) to reach OD_{600}~0.1. Next, 100 μl of diluted lysate of T4 phages was added to all three flasks that contained 10 ml of refreshed bacterial cultures (to reach a final concentration of 50,000 PFU in the flask). 200 μl of the suspension was taken at each time point from all the flasks, transferred to 200 μl chloroform and immediately vortexed (the bacteria were killed by the chloroform and phages were released from the bacterial cells). The samples were centrifuged (8,000, 2 minutes; Eppendorf MiniSpin, Germany) to spin down the chloroform in the tubes. 10 μl of the upper aqueous layer with phages was taken to prepare a series of dilutions in TM buffer (10 mM Tris-HCl with 10 mM $MgSO_4$, pH = 7.4) to obtain around 20 plaques in a single droplet. Just before pipetting the droplets from each dilution, 4 ml of top LB agar (consisting of LB medium and 0.75% agar) was mixed with 200 μl of refreshed control culture of *E. coli* BL21 (which had not been exposed to phages) and immediately poured onto the plate with 25 ml of bottom agar. After solidification, phage titration was performed on the plates (with bottom and top agar) by pipetting droplets of 5 μl volume. At least 5 technical replicates from each dilution were made. At the same time point 50 μl of suspension from the flask was transferred to TM buffer, diluted and pipetted onto the bottom agar. This was done to determine the number of bacteria in the sample to be sure that all available bacteria had been infected by

the phages. All the plates with droplets were left to dry. The plates were incubated for 24 h: those with phages at 37°C and those with bacteria at 30°C. The plaques and the bacteria were counted from each droplet. Plaque forming units (PFU) were correlated with the number of active phages within the sample. One biological replicate of the experiment was performed.

11. Induction of the SOS response

Unfavorable environmental conditions can force cells to induce a DNA repair system that helps them to survive sudden damage to DNA[10]. We checked whether exposure of *E. coli* cells to ZnO nanorods and rounded nanoparticles could induce SOS response.

Damage to DNA leads to the SOS response in bacteria that triggers the induction of prophages (a latent form of bacteriophage that is integrated into bacterial DNA). Induction of prophages means transformation from a lysogenic to a more aggressive, lytic cycle, during which numerous copies of progeny phages are created and released from the disrupted bacterial cells[11]. The level of DNA damage is reflected in the number of released phages, since the higher the stress, the higher the number of progeny phages. Therefore, in our experiments, we simultaneously analyzed the number of phages and the number of living bacteria. The number of bacteria decreased during exposure to ZnO nanorods and nanoparticles. The number of phages divided by the number of bacteria ((PFU/CFU) per ml) is presented (**Supplementary Fig. S18**). Mitomycin C, a drug that causes crosslinking of DNA and inhibits replication, was used as a marker that induced a high SOS response. The higher induction of the SOS signal in samples with nanostructures, in comparison to the control sample, can be related to a higher probability of piercing of the bacteria during collisions. The disintegration or increase impermeability of the cell wall can cause an influx of external solvent to the interior of the cell and can induce stress. This could result in a higher induction of prophage. The SOS response in the case of samples with ZnO nanorods (NR) and nanoparticles (NP) is maintained at a

relatively low level in comparison to mitomycin C stimulation. This proved that DNA damage was not significant.

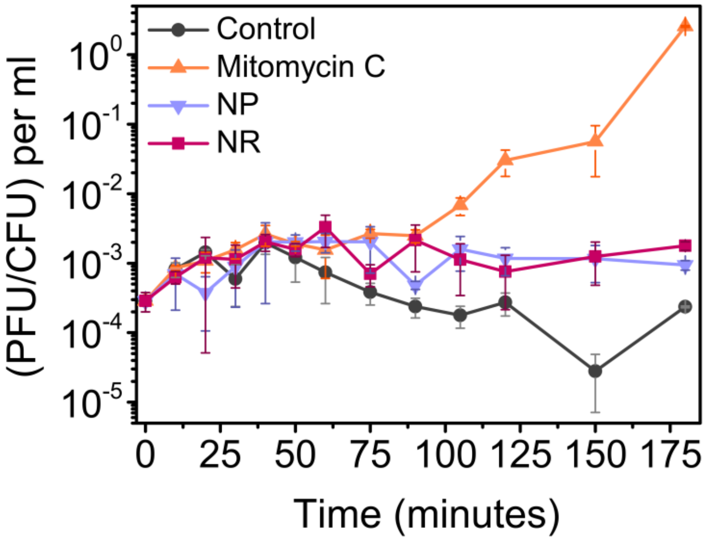

Supplementary Figure S18. Induction of SOS signal in *E. coli* K12 MG 1655 (prophage λ PaPa). The difference in number of plaque forming units (PFU) divided by the number of the bacteria (CFU) for control *E. coli* (marked as Control), *E. coli* cells exposed to mitomycin C (marked as Mitomycin C), *E. coli* cells after exposure to ZnO nanorods (marked as NR0 and *E. coli* cells after exposure to ZnO nanoparticles (marked as NP). Error bars show standard error of the mean (s.e.m.).

Procedure: In this experiment, *Escherichia coli* K12 MG1655 (prophage λ PaPa) and *Escherichia coli* K12 MG1655 were used (obtained from the Department of Molecular Genetics of Bacteria, Faculty of Biology, University of Gdańsk, Poland). *E. coli* K12 MG1655 (prophage λ PaPa) was used to determine the number of phages. *E. coli* K12 MG1655 was used to evaluate the number of bacteria upon exposure to ZnO nanorods and nanoparticles. Two overnight cultures were prepared by inoculation of a single colony into LB for (37 °C, 180 rpm). On the day of the experiment, the bottom agar was prepared for phage titration by pouring 25 ml of LB agar medium into sterile plastic Petri dishes. Just before the experiment, the cultures were refreshed by diluting the suspension of bacteria in LB medium (volume ratio 1:100) to reach OD~0.1. The refreshed bacterial culture of *E. coli* K12 MG1655 (prophage λ PaPa) and *E. coli*

K12 MG1655 was divided into 4 sterile flasks (8 flasks in total, 4 for each strain (control, mitomycin C, NR, NP) 24 ml per flask). In the case of *E. coli* K12 MG1655 (prophage λ PaPa) the number of phage plaques (PFU) was determined, whereas for *E. coli* K12 MG1655 the number of colony forming units (CFU) was analyzed.

1 ml of LB medium was added to the control flasks. Mitomycin C (Sigma Aldrich), diluted in 1 ml of LB medium, was added to the second flask with the suspension of bacteria to obtain a final concentration of 0.5 μg ml^{-1}. To the third and fourth flask ZnO nanorods and spherical nanoparticles (suspended in 1 ml of LB medium) were added respectively, in concentrations of 1 mg ml^{-1}.

Every half hour 200 μl of all four suspensions of *E. coli* MG1655 (prophage λ PaPa) were taken from all flasks and added to 200 μl of chloroform and immediately vortexed (the bacteria were killed by the chloroform and phages were released from the bacterial cells). The samples were centrifuged (6,000 rpm, 5 minutes; Eppendorf MiniSpin, Germany) to obtain a chloroform layer at the bottom of the tubes. For all samples, 10 μl of the upper aqueous layer with phages was taken to prepare a series of dilutions (to obtain around 20 plaques in a single droplet). Just before pipetting the droplets from each dilution, 4 ml of top LB agar (consisting of LB medium and 0.75% agar) was mixed with 200 μl of the refreshed, culture of control *E. coli* K12 MG1655 and immediately poured into the plate with 25 ml of bottom agar. After solidification, phage titration was performed on the plates (with bottom and top agar) by pipetting droplets with a volume of 5 μl (at least 5 technical replicates) for each dilution.

At the same time points, 200 μl were taken from all four suspensions of *E. coli* MG1655 and transferred into tubes. 100 μl of each sample was used for further dilutions. Titration of the bacteria was performed on the plates with bottom agar by pipetting droplets with a volume of 10 μl. For each dilution at least 5 technical replicates were performed.

All the plates (for determination of the number of bacteria and phages) with droplets were left

to dry completely. The plates with *E. coli* K12 MG1655 (prophage λ PaPa) were incubated at 37 °C for 24 h. The plates with *E. coli* K12 MG1655 were incubated at 30 °C for 24 h (the temperature was decreased in order to reduce the size of the growing colonies). The plaques and the bacteria were counted from each droplet. Plaque forming units (PFU) and colony forming units (CFU) were directly correlated with the number of active phages and living bacteria respectively within the sample. Two biological repeats of the experiment were performed.

12. References

1. Matuła, K. *et al.* Influence of nanomechanical stress induced by ZnO nanoparticles of different shapes on the viability of cells. *Soft Matter* **12,** 4162–9 (2016).

2. Legendre, D., Zenit, R., Daniel, C. & Guiraud, P. A note on the modelling of the bouncing of spherical drops or solid spheres on a wall in viscous fluid. *Chem. Eng. Sci.* **61,** 3543–3549 (2006).

3. Lan, G., Wolgemuth, C. W. & Sun, S. X. Z-ring force and cell shape during division in rod-like bacteria. *Proc. Natl. Acad. Sci. U. S. A.* **104,** 16110–5 (2007).

4. Yoshimura, H. N., Molisani, A. L., Narita, N. E., Manholetti, J. L. A. & Cavenagho, J. M. Mechanical properties and microstructure of zinc oxide varistor ceramics. *Mater. Sci. forum* **530–31,** 408–413

5. Gumbart, J. C., Beeby, M., Jensen, G. J. & Roux, B. Escherichia coli peptidoglycan structure and mechanics as predicted by atomic-scale simulations. *PLoS Comput. Biol.* **10,** e1003475 (2014).

6. Sneddon, I. N. The relation between load and penetration in the axisymmetric boussinesq problem for a punch of arbitrary profile. *Int. J. Eng. Sci.* **3,** 47–57 (1965).

7. Cayley, D. S., Guttman, H. J. & Record, Jr., M. T. Biophysical characterization of changes in amounts and activity of Escherichia coli cell and compartment water and turgor pressure in response to osmotic stress. *Biophys. J.* **78,** 1748–1764 (2000).

8. McDonald, J. H. *Handbook of biological statistics, Second edition*. (2009).

9. Feiner, R. *et al.* A new perspective on lysogeny: prophages as active regulatory switches of bacteria. *Nat. Rev. Microbiol.* **13,** 641–650 (2015).

10. Michel, B. After 30 years of study, the bacterial SOS response still surprises us. *PLoS Biol.* **3,** e255 (2005).

11. Janion, C. Inducible SOS response system of DNA repair and mutagenesis in Escherichia coli. *Int. J. Biol. Sci.* **4,** 338–44 (2008).
